# Sweat Gland Organoids Originating from Reprogrammed Epidermal Keratinocytes Functionally Recapitulated Damaged Skin

**DOI:** 10.1002/advs.202103079

**Published:** 2021-09-26

**Authors:** Xiaoyan Sun, Jiangbing Xiang, Runkai Chen, Zhijun Geng, Lintao Wang, Yiqiong Liu, Shuaifei Ji, Huating Chen, Yan Li, Cuiping Zhang, Peng Liu, Tao Yue, Lei Dong, Xiaobing Fu

**Affiliations:** ^1^ Research Center for Tissue Repair and Regeneration affiliated to the Medical Innovation Research Department and 4th Medical Center PLA General Hospital and PLA Medical College PLA Key Laboratory of Tissue Repair and Regenerative Medicine and Beijing Key Research Laboratory of Skin Injury Repair and Regeneration Research Unit of Trauma Care Tissue Repair and Regeneration Chinese Academy of Medical Sciences 2019RU051 Beijing 100048 P. R. China; ^2^ Bioengineering College of Chongqing University Chongqing 400044 P. R. China; ^3^ Department of General Surgery Chinese PLA General Hospital 28 Fu Xing Road Beijing 100853 P. R. China; ^4^ State Key Laboratory of Pharmaceutical Biotechnology School of Life Sciences Nanjing University Nanjing Jiangsu 210023 China; ^5^ Department of Biomedical Engineering School of Medicine Tsinghua University Haidian District Beijing 100084 China; ^6^ School of Mechatronic Engineering and Automation Shanghai University Shanghai 200444 China; ^7^ Shanghai Institute of Intelligent Science and Technology Tongji University Shanghai 200092 China

**Keywords:** organoids, regeneration, reprogramming, skin, stem cells, sweat glands

## Abstract

Restoration of sweat glands (SwGs) represents a great issue in patients with extensive skin defects. Recent methods combining organoid technology with cell fate reprogramming hold promise for developing new regenerative methods for SwG regeneration. Here, a practical strategy for engineering functional human SwGs in vitro and in vivo is provided. First, by forced expression of the ectodysplasin‐A in human epidermal keratinocytes (HEKs) combined with specific SwG culture medium, HEKs are efficiently converted into SwG cells (iSwGCs). The iSwGCs show typical morphology, gene expression pattern, and functions resembling human primary SwG cells. Second, by culturing the iSwGCs in a special 3D culturing system, SwG organoids (iSwGOs) that exhibit structural and biological features characteristic of native SwGs are obtained. Finally, these iSwGOs are successfully transplanted into a mouse skin damage model and they develop into fully functioning SwGs in vivo. Regeneration of functional SwG organoids from reprogrammed HEKs highlights the great translational potential for personalized SwG regeneration in patients with large skin defects.

## Graphical abstract

An engineering strategy is shown to generate organoids from reprogrammed human epidermal keratinocytes (HEKs) for the regeneration of human sweat glands (SwGs). The technology enables efficient conversion of HEKs into SwG cell lineages and allows a large‐scale production of tissue‐relevant organoids for personalized SwG regeneration in patients with large skin defects.

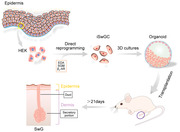

## 1 Introduction

Sweat glands (SwGs), tiny coiled tubular skin appendages, are essential for human survival. They help to balance the body temperature in response to hot climate and exercise. They also function as excretory epidermal appendages, responsible for the body's fluid and electrolyte homeostasis.^[^ ^1^ ^]^ However, for patients with large skin defects, the severe injury and following hypertrophic scar formation usually causes disruption to the overall skin architecture and loss of appendages. Loss of SwGs can dramatically decrease a patient's quality of life because he may have problems with working and exercising and be at higher risk for heat‐related illnesses such as hyperthermia, orstroke, or even death. Therefore, restoring SwGs represents a significant issue in the functional wound care of extensive skin defects.

SwGs are maintained by stem and progenitor cells within the ducts and coiled glands.^[^ ^2^ ^]^ For large wounds, tissue damage often causes an overall reduction in local stem cells, and progenitor cells within residual SwGs show limited regenerative capacities and respond inefficiently to tissue damage cues.^[^ ^2^ ^]^ As a result, de novo SwG regeneration rarely occurs during wound healing. To improve treatment outcomes, autologous or allogeneic transplant has become a complementary choice, but application of this strategy is still restricted owing to the shortage of donor skin and low in vitro expansion capacity of primary SwG cells. To solve these problems, new methods for generating functional mature SwG cells with sufficient efficiency are in great demand. Recent studies have demonstrated the potential conversion of autologous somatic cells, such as the epidermal progenitors^[^ ^3^ ^]^ or mesenchymal stem cells,^[^ ^4^ ^]^ into SwG lineages through reprogramming manipulations. According to the current lineage reprogramming strategy, which usually involves enforced expression of lineage determination factors or specific signaling pathway elements, the reprogramming process is generally considered to work better when starting cells share similar embryonic germ‐layer origins.^[^ ^5^ ^]^ SwGs are derived from an epidermal bud of CK5‐expressing progenitors,^[^ ^2^, ^6^ ^]^ sharing the same origin as epidermal keratinocytes that can be readily maintained and propagated in vitro.^[^ ^7^ ^]^ Several studies have suggested an unanticipated level of cellular plasticity within epidermal keratinocytes, resulting in cell‐fate flexibility during wound repair.^[^ ^8^, ^9^, ^10^, ^11^, ^12^ ^]^ Therefore, if epidermal keratinocytes could be converted into SwG cells, combined with their easy accessibility, they could be used as a source to meet the clinical need of producing large amounts of fully functional SwG cells with robust engrafting ability. Ectodysplasin‐A (EDA) signaling has a key role in SwG initiation and development.^[^ ^13^, ^14^ ^]^ Our previous study demonstrated the conversion of bone marrow‐derived mesenchymal stem cells into SwG cells via forced EDA gene expression,^[^ ^4^ ^]^ which suggests that EDA can act as an intrinsic factor to re‐establish a SwG cell identity during SwG reprogramming. Also, fully defined conditions must be used, such as a SwG‐specific culture medium, to enhance the efficiency of reprogramming and accelerate maturation of the converted cells.^[^ ^15^ ^]^

Full‐thickness wounds often involve the ablation of epidermal appendages and also irrevocable damage to dermal connective components. Accordingly, exogenous or endogenous stem cells at the wound bed fail to form SwGs owing to the absence of a glandular permissive tissue environment. To overcome these challenges, recent organoid technologies could be exploited. Organoids developed in the artificial in vitro conditions retain the organ identity,^[^ ^16^, ^17^ ^]^ and more importantly, the formation of organoids also recapitulate a 3D in vivo‐like microenvironment that may well compensate for the lack of structural support for tissue formation in the damaged extracellular matrix environment when used as xenografts.^[^ ^18^, ^19^ ^]^ Moreover, patient‐specific organoid engineering by using reprogramming cells for regenerative therapy has progressed.^[^ ^20^ ^]^ This allows for cost‐effective generation of transplantable organoids from individual patients without requiring ex vivo expansion of difficult‐to‐preserve primary cells such as SwG cells. The establishment of somatic reprogramming‐derived organoids also provides a controllable system, whereby these tissue‐committed organoids in 3D cultures might re‐establish a gland‐permissive environment in the wound bed. This in turn will enable organotypic reconstructions resembling that of SwG organogenesis, and it will avoid the uncertainty of stem cell transplantation during wound healing. More importantly, significantly different from large organs, for SwG organoids, the initial size is close to that of native sweat buds during SwG development, so their transplantation for regeneration is highly feasible.

In this study, we sought to regenerate functional SwGs by using reprogrammed human epidermal keratinocytes (HEKs) via a stepwise strategy. We initially reprogramed HEKs into SwG cells by forced expression of EDA in combination with specific culture conditions. We then established SwG organoids from the reprogrammed HEKs, and finally we transplanted the organoids into a mouse model for the regeneration of functioning SwGs in vivo. With this strategy, it is possible to generate human SwG organoids at a sufficiently large scale for clinical use.

## 2 Results

### 2.1 Activation of *β*_2_‐AR Signaling Increases Stemness in HEKs

We initially tested the possibility to induce the conversion of epidermal keratinocytes into SwG cells by using EDA as the lineage‐specific core regulator. Following the transduction of lentiviral vectors expressing EDA and GFP within HEKs, the EDA‐GFP^+^ population was FACS sorted (Figure S1a, Supporting Information). Overexpression of EDA alone did not alter the HEK morphology (Figure S1b, Supporting Information) or upregulate the SwG‐associated gene expression (Figure S1c, Supporting Information). Despite RNA‐seq analysis showing a similar transcriptional expression profile (Figure S1d, Supporting Information), the differentially expressed genes (DEGs) in HEK‐EDA versus HEK cells were mainly enriched in myoblast differentiation, cell projection, extracellular matrix organization, and gland morphogenesis, potentially involved in SwG development (Figure S2a, Supporting Information). Therefore, EDA alone was not sufficient to reprogram HEKs into SwG cells.

Given the roles of environmental conditions in facilitating somatic reprogramming,^[^ ^21^ ^]^ we next tested whether HEK‐EDA cells could be converted into SwG cells by an optimized SwG induction medium (SGM) containing epidermal growth factor (EGF) and basic fibroblast growth factor (bFGF).^[^ ^22^ ^]^ Culture in SGM induced some SwG‐like morphological changes in HEK‐EDA cells. Lumenized networks lined by HEK‐EDA cells were observed in cell cultures (Figure S1b, Supporting Information). Moreover, these HEK‐EDA cells expanded robustly and were immunopositive for SwG markers (Figure S2b, Supporting Information). However, although long‐term exposure to SGM upregulated CK18, aquaporin 5 (AQP5) and *α*‐SMA transcription in HEK‐EDA cells (Figure S2c, Supporting Information), only a small proportion of the reprogrammed cells could be induced to express the ductal (CK5^+^/CK10^+^, 5.01±1.47%), luminal (CK18^+^/AQP5^+^, 6.31±1.64%) or myoepithelial (CK5^+^/*α*‐SMA^+^, 3.37±0.87%) markers (Figure S2d,e, Supporting Information), which indicates that additional treatments were required to boost the conversion efficiency of induced SwG cells (iSwGCs).

Cells with enhanced stemness have been reported to be more amenable to reprogramming,^[^ ^23^, ^24^, ^25^ ^]^ so we examined whether greater stemness could drive cell fate conversion of HEKs toward SwG cells more efficiently. Following FACS sorting based on skin stem‐cell marker LGR5 expression (LGR5^hi^ vs LGR5^lo^, Figure S3a, Supporting Information), we transduced LGR5^hi^ or LGR5^lo^ subpopulations of HEKs and found that EDA overexpression in LGR5^hi^ cells conferred significantly higher levels of BMP5, CEA, CK18, CK19, CK5, *α*‐SMA, and AQP5 expression when cells were cultured in SGM (Figure S3b,c, Supporting Information). This finding suggested that increasing the stemness of HEKs could promote the reprogramming efficiency.

**Figure 1:**
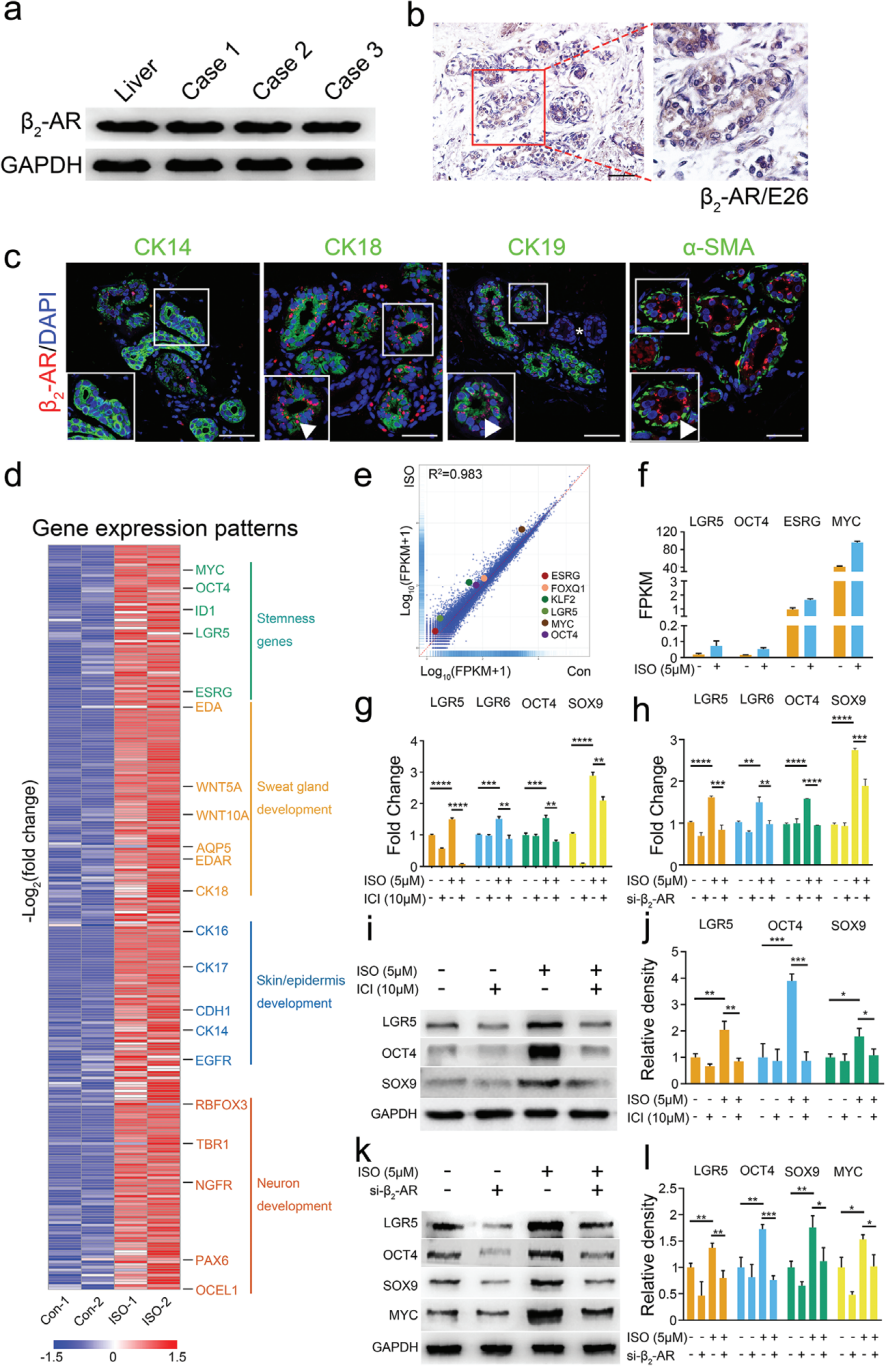
Activation of *β*_2_‐AR signaling induced stemness acquisition in HEKs. a) Western blot analysis of *β*_2_‐AR expression in human skin samples. Human liver tissues were used as positive controls (*n* = 3); b) Immunohistochemistry of *β*_2_‐AR in human developing glands at embryo day 26 (E26). Scale bar represents 100 µm; c) Representative immunofluorescence showing the co‐labeling of *β*_2_‐AR with duct marker CK14, luminal markers CK18, CK19, and myoepithelial marker *α*‐SMA in human mature SwGs. Boxed area is magnified to see *β*_2_‐AR typically restricted in the CK18^+^ or CK19^+^ luminal cells (white arrows). Star showing SwG ducts. Scale bar represents 25 µm; d) Heatmap illustrating the genes involved in stemness regulation, skin development, gland organogenesis, and neuron development in HEKs with or without ISO treatment (FDR ≤ 0.05 and |FC| ≥ 2); e) Scatter plot of RNA‐seq transcriptome analyses between HEKs treated with or without ISO. Stemness‐associated genes were enriched in ISO‐treated HEKs versus controls (colored dots); f) Expression of stemness‐associated genes in ISO‐treated and untreated HEKs; g) For *β*_2_‐AR blockage, starved HEKs were pre‐treated with 10 µm ICI‐118551 for 3 h. The cells were subsequently incubated in the presence or absence of 5 µm ISO for an additional 24 h. Then, relative mRNA expression levels of LGR5, LGR6, OCT4, SOX9 was assessed by quantitative RT‐PCR (*n* = 3). The results were normalized to GAPDH expression; h) qRT‐PCR analysis showing LGR5, LGR6, OCT4, SOX9 expression in HEKs and si‐*β*_2_‐AR‐treated HEKs with or without ISO (*n* = 3); i) Western blot analysis was performed to investigate the protein levels of LGR5, OCT4, SOX9 in HEKs after switched to the medium containing 0, 5 µm ISO with or without ICI‐118551 (*n* = 3); j) Quantitative western blot analysis of protein expression for LGR5, OCT4, and SOX9. The densitometric values were qualified with Image J software and the relative density (compared with untreated HEKs) of the indicated protein was calculated; k) Western blot analysis of LGR5, LGR6, OCT4, SOX9 expression levels in HEKs and si‐*β*_2_‐AR‐treated HEKs with or without ISO treatment (*n* = 3); l) Qualification of LGR5, LGR6, OCT4, SOX9 immunoblots was performed using Image J software. GAPDH was used as internal loading control. Data are mean ± SD. *p < 0.05, ** *p* < 0.01, *** *p* < 0.001, **** *p* < 0.0001.

As a peripheral neuroendocrine‐control organ,^[^ ^26^, ^27^ ^]^ SwGs normally express *β*_2_‐adrenoceptors (*β*_2_‐ARs) as well as acetylcholine (ACh) receptors in the secretory domain.^[^ ^28^, ^29^ ^]^ In the present study, we detected *β*_2_‐AR expression in human adult and fetal skin biopsies (**Figure** **1a**,b), which was restricted progressively from ductal cells to the secretory domain of developing glands in fetal specimens from a 26‐week embryo (Figure 1b). Further analysis demonstrated that *β*_2_‐AR was strongly expressed in the CK18^+^/CK19^+^ luminal glandular cells in adult SwGs (Figure 1c and Figure S4a,b, Supporting Information). Moreover, functional sweating could be induced in mouse paws upon *β*_2_‐AR activation by intradermal injection of 5 µg mL^−1^ isoproterenol (ISO, Figure S4c, Supporting Information). The expression of *β*_2_‐AR is particularly important in accordance with our recent findings that *β*_2_‐AR signaling influenced cellular plasticity of epidermal keratinocytes^[^ ^30^ ^]^ and increased stemness with restoration of stem cell properties within differentiated cells.^[^ ^31^ ^]^ Therefore, activating *β*_2_‐AR would probably have the same effect on increasing the stemness in HEKs. To address this, we used the *β*_2_‐AR agonist ISO to stimulate HEKs and found significant changes in RNA‐seq results, identifying a total of 5314 known DEGs, 2823 upregulated and 2491 downregulated, in ISO‐treated HEKs (Figure S5a, Supporting Information, false discovery rate [FDR] ≤ 0.05 and fold change |FC| ≥ 2.0). Moreover, analysis of global transcriptomic changes showed that some important signaling pathways involved in regulation of stem cell self‐renewal, including MAPK,^[^ ^32^, ^33^ ^]^ JAK‐STAT,^[^ ^34^ ^]^ Hippo,^[^ ^35^ ^]^ and FoxO^[^ ^36^ ^]^ pathways, were upregulated in ISO‐treated HEKs (Figure S5b, Supporting Information). Comparative bioinformatics further revealed that a panel of stemness‐related genes including LGR5, OCT4, ESRG, MYC, KLF2, and FOXQ1were significantly enriched (*p* < 0.05, [FDR] ≤ 0.05, and |FC| ≥ 2) in HEKs after ISO stimulation (Figure 1d–f). The RNA‐seq results were verified by quantitative PCR (qPCR) or western blot analysis of some key genes, including epidermal progenitor associated markers LGR5^[^ ^37^ ^]^ and LGR6^[^ ^38^ ^]^ and pluripotency‐related genes OCT4 and SOX9. Consistently, ISO treatment upregulated LGR5, LGR6, OCT4, and SOX9 expression in HEKs (Figure 1g–l). The effect of ISO was additionally determined in HaCaT keratinocytes (Figure S6a–c, Supporting Information). Importantly, *β*_2_‐AR inhibition by ICI‐118551 (Figure 1g,i,j) or *β*_2_‐AR siRNAs (Figure 1h,k,l) attenuated the effect of ISO, which further demonstrates the importance of *β*_2_‐ARs in upregulating the stemness of HEKs.

Interestingly, gene ontology (GO) analysis of the upregulated 2366 transcripts indicated gene sets involved in epidermal development programs (Figure S7a, Supporting Information). Also, ISO stimulation enriched genes related to gland morphogenesis and development in HEKs (Figure S7b, Supporting Information). Of these, BMP5, EDA, EDAR, FGF18, WNT5A ligand and its downstream target ID1 were expressed at relatively higher levels as compared to controls (Figure S7c, Supporting Information). Given that BMPs, WNT5A and WNT10A are critical in SwG specification, causing a switch from hair follicle to SwG fate at the epithelium placode stage,^[^ ^6^ ^]^ their expression differences were then confirmed on qPCR in HEKs with or without ISO treatment (Figure S7d, Supporting Information). Upregulation of some specific SwG markers was also observed in ISO‐treated HEKs (Figure S7e,f, Supporting Information). However, inhibition of *β*_2_‐AR with ICI‐118551 reversed the ISO‐induced BMP5, EDA, CK5, CK18, CK19, CEA expression (Figure S7e,f, Supporting Information), so activation of *β*_2_‐AR signaling plays a role on priming HEKs for the SwG fate.

Altogether, these data showed that stimulating *β*_2_‐AR with ISO significantly increased the stemness of HEKs and might have the potential to promote the efficiency of the cell‐fate conversion to SwG cells via EDA reprogramming.

### 2.2 Conversion of HEKs into iSwGCs by Combining *β*_2_‐AR Activation, EDA Overexpression and SGM Culturing

**Figure 2:**
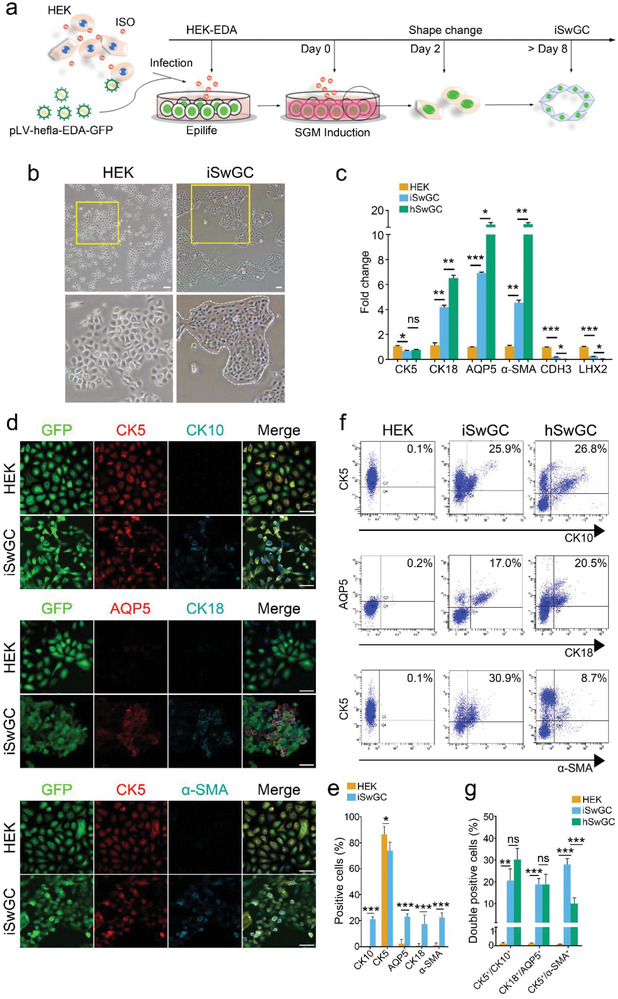
Reprogramming of HEKs into SwG cells by combining the stimulation of *β*_2_‐AR, forced transgenic expression of EDA and SGM culture. a) Scheme of ISO‐based reprogramming procedure. ISO‐treated HEKs were transduced with EDA and plated in Epilife. Then cells were transferred into SGM supplemented with 5 µm ISO (Day 0) and cultured for indicated days; b) Phase contrast images showing the morphological changes of iSwGCs in optimized SGM containing ISO. Scale bar = 100 µm. Insets, higher magnification of the boxed areas; c) qPCR analysis of transcriptional expression of CK5, CK18, AQP5, *α*‐SMA, and hair follicle‐specific genes LHX2, CDH3 in HEKs, and iSwGCs after 8 days of induction. Primarily isolated SwG cells from human skin samples (hSwGCs) were used as positive controls. The genes showing significant change in PCR array assay are presented; d) Representative immunofluorescence of CK5, CK10, AQP5, CK18, and *α*‐SMA in HEKs, and iSwGCs at day 20 after SGM treatment with ISO. Scale bar = 50 µm; e) Percentages of CK5^+^, CK10^+^, AQP5^+^, CK18^+^, and *α*‐SMA^+^ cells in HEKs and iSwGCs calculated according to the immunostaining. Quantification was done with 5 randomly selected microscopy fields from each of the 3 independent experiments; f) FACS analysis showing the cell fractions labeled with antibodies against CK5, CK10, AQP5, CK18, and *α*‐SMA in HEKs, iSwGCs and native hSwGCs. g) Proportions and absolute numbers of the CK5^+^/CK10^+^, AQP5^+^/CK18^+^, and CK5^+^/*α*‐SMA^+^ cell population in HEKs, iSwGCs, and hSwGCs. *n* = 3. Data are mean ± SD and analyzed by two‐tailed *t*‐tests, * *p* < 0.05, ** *p* < 0.01, *** *p* < 0.001. ns, not significant.

In light of the above results, we designed the strategy in **Figure** **2a** to systemically reprogram HEKs into iSwGCs by combining stimulation with *β*_2_‐AR, forced transgenic expression of EDA, and SGM culture. Reprograming quickly promoted the morphological changes in HEK‐EDA cells at 2 days after ISO stimulation with SGM treatment and generated GFP‐positive cells with SwG‐like morphology (Figure 2b). After 8 days of induction, RNA expression profiling by qRT‐PCR revealed a significant increase in levels of key SwG genes including CK18, AQP5, and *α*‐SMA but not CK5 in iSwGCs as compared with primary isolated HEKs (Figure 2c). In contrast, hair follicle‐related genes were downregulated (Figure 2c), which indicated that most HEK‐converted cells engaged a SwG fate. We verified the marker expression of iSwGCs by immunostaining (Figure 2d,e), and western blot analysis further showed that the upregulation of SwG‐specific genes in iSwGCs in response to ISO indeed depended on the activation of *β*_2_‐ARs (Figure S8a,b, Supporting Information). Further FACS analysis in Figure 2f,g suggested that the iSwGCs were heterogeneous in that ≈20.53 ± 5.4% of the reprogrammed cells had adopted a sweat duct cell phenotype (CK5^+^/CK10^+^), 18.73 ± 2.8% adopted a luminal phenotype (CK18^+^/AQP5^+^), and 27.97 ± 2.73% adopted a myoepithelial phenotype (CK5^+^/*α*‐SMA^+^).

To further test the reliability of the induction procedure, we used a Tet‐on CRISPR system for conditional EDA activation to direct the fate conversion of the HaCaT keratinocyte cell line. A gRNA vector containing a GFP reporter was designed to target upstream of the EDA transcriptional start site, and a tetracycline/doxycycline‐responsive element (TRE) was used to control the expression of dCas9 effectors (dCas9‐E).^[^ ^39^ ^]^ HaCaT cells were transduced with the gRNA and dCas9‐E vectors, then treated with doxycycline (dox) (Figure S9a,b, Supporting Information). After 2 days’ exposure to SGM supplemented with ISO, the expression of dCas9‐E and EDA targeting guides reprogrammed HaCaT cells into SwG‐like cells (Figures S9c–f and S10a–d, Supporting Information).

Taken together, these data demonstrate that we developed an efficient two‐step strategy to reprogram HEKs into SwG cells.

### 2.3 iSwGCs Possessed Functional Characteristics of Human SwG Cells

**Figure 3:**
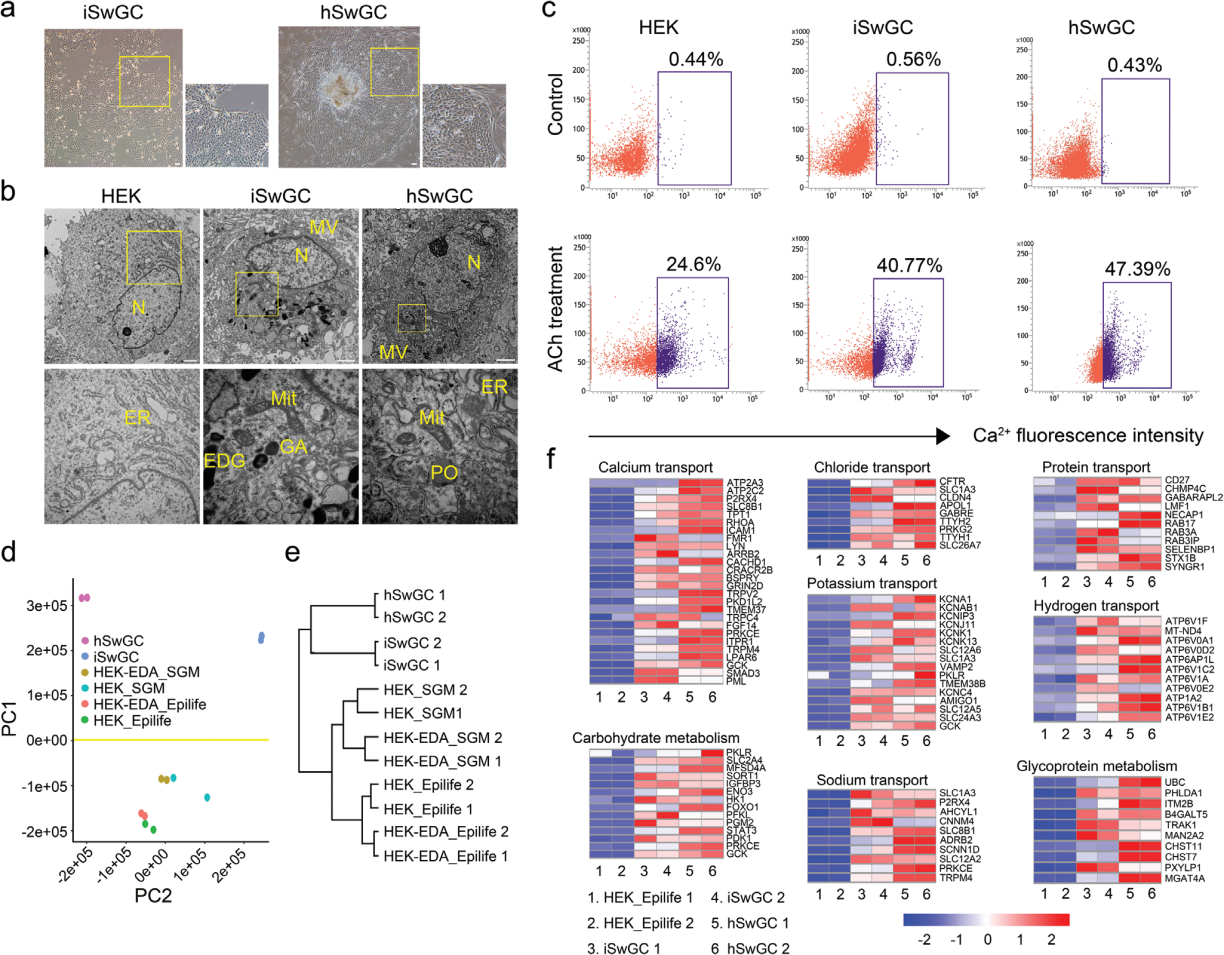
iSwGCs possessed functional characteristics of hSwGCs. a) Phase‐contrast image of iSwGCs and isolated hSwGCs. Scale bar = 100 µm; b) Ultrastructure analysis of HEKs, iSwGCs, and hSwGCs. Note an ultrastructure with increased subcellular organelles and abundant secretory electron dense granules in iSwGCs and hSwGCs compared to primary HEKs. Scale bars = 2000 nm. Insets, higher magnification of the boxed areas. N = Nuclear; Mit = Mitochondria, MV = Microvilli, ER = Endoplasmic reticulum, PO = Peroxisomes, EDG = Electron dense granules, GA = Golgi apparatus; c) FACS analysis of Ca^2+^ flux in Ach‐treated HEKs, iSwGCs, and hSwGCs after loading cells with calcium orange, a sensitive Ca^2+^ indicator (*n* = 3); d) PCA, and e) hierarchical clustering analysis of the global gene expression patterns within HEKs, Epilife‐treated HEK‐EDA cells, SGM‐treated HEKs, SGM‐treated HEK‐EDA cells, iSwGCs, and hSwGCs during SwG reprogramming; f) Heatmap depicting the representative GO terms of gene expression in iSwGCs and hSwGCs compared to HEKs. FDR ≤ 0.05 and |FC| ≥ 2.

Given that the iSwGCs were similar in morphology to native SwG cells (**Figure** **3a**), we further determined whether these cells possessed the functional properties characteristic of native SwG cells. First, ultrastructure analysis showed a significantly increased number of subcellular organelles in iSwGCs as compared with untreated HEKs, and the mitochondria of iSwGCs exhibited the same normal cristae structure as that of human SwG cells (hSwGCs, Figure 3b). Second, given that calcium influx from the extracellular space is essential for SwG function and sweating,^[^ ^40^ ^]^ we measured the intracellular Ca^2+^ signals by using an orange fluorescent calcium indicator and found a similar level of cytoplasmic free Ca^2+^ intensity in iSwGCs as in hSwGCs (Figure 3c). Third, we compared the transcriptomes of iSwGCs with their parental HEKs and the primary hSwGCs by RNA‐seq and found that 1) the iSwGCs expressed major SwG genes, channel genes, and carbohydrate metabolic genes (Figure S11, Supporting Information); 2) heatmap analysis showed a significant difference between iSwGCs and parental HEKs and a high similarity between iSwGCs and control hSwGCs (Figure S11, Supporting Information); 3) genes critical to gland development, actin cytoskeleton organization and regulation, and cell secretion were also upregulated in iSwGCs (Figure S11, Supporting Information); and 4) principle component analysis (PCA) and hierarchical clustering analysis revealed that iSwGCs generated from our reprogramming procedure were clustered with hSwGCs but separated from the untreated HEKs and HEK‐EDA cells without SGM treatment in the PC1 dimension (Figure 3d,e); and 5) As previously reported,^[^ ^2^ ^]^ iSwGCs showed the gene expression patterns of functional SwGs for a set of genes involved in iron transport, protein transport, carbohydrate metabolism, and glycoprotein metabolism (Figure 3f).

Together, these results indicate that iSwGCs showed gene expression profiles and functional properties characteristic of their native counterparts.

### 2.4 Establishment of Human SwG Organoids from iSwGCs

**Figure 4:**
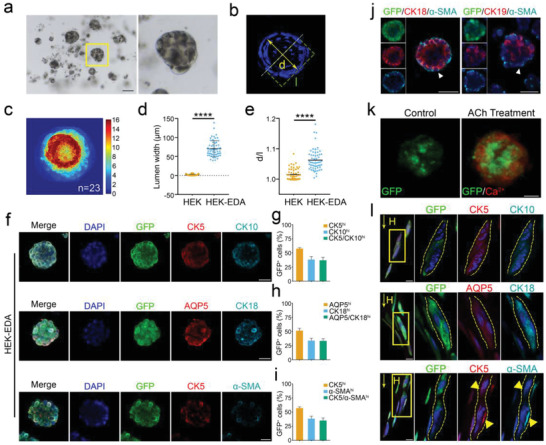
Establishment of human SwG organoids from iSwGCs. a) Representative phase contrast images showing iSwGO cultures. Scale bar = 100 µm; b) Representative images of lumen‐containing organoids derived from iSwGCs. d and l represent the diameter (lumen width) and long axis of the lumen, respectively; c) Signal distribution acquired by confocal microscopy showing the quantification of luminal‐containing organoids derived from reprogrammed HEKs (*n* = 23). Organoids with similar size were analyzed independently from 3 biological replicates; d,e) Scatter plots representing the features of iSwGOs. HEKs cultured under the same 3D condition were controls; f) Immunofluorescence assay of ductal markers CK5 and CK10, luminal markers AQP5 and CK18, and myoepithelial markers CK5 and *α*‐SMA in iSwGOs. The iSwGOs were obtained at passages 2–4 after the initiation of 3D culture. Scale bar = 50 µm; g–i) Percentages of CK5^+^/CK10^+^‐, AQP5^+^/CK18^+^‐, and CK5^+^/*α*‐SMA^+^‐expressing organoids in each GFP‐positive population were shown. Quantifications involved > 100 organoids from 3 independent experiments. Data are mean ± SD; j) Immunofluorescence co‐staining of *α*‐SMA with CK18 or CK19 in iSwGOs generated from CD49f^hi^CD29^hi^ cells. Scale bar = 75 µm; k) Fluorescence live cell imaging of intracellular Ca^2+^ activity in iSwGOs after ACh addition. Scale bar = 25 µm, *n* = 3; l) Immunofluorescence analysis of expression of ductal markers CK5 and CK10, luminal markers AQP5 and CK18 and myoepithelial markers CK5 and *α*‐SMA in tubular structures generated from iSwGOs in response to bFGF gradients. Scale bar = 25 µm.

To establish organoid cultures in vitro, single iSwGCs were embedded in matrigel and cultured in SGM induction medium. Under this situation, single cells initially formed a solid ball and self‐organized into monoclonal spheres within 2 weeks (**Figure** **4a**). The organoids with distinctive layers could be consistently passaged and stably maintained under the 3D culture condition for more than 3 months. Also, ISO added in the medium could support the growth of large organoids in iSwGCs (Figure S12a,b, Supporting Information). These iSwGC‐derived organoids (iSwGOs) could expand to 200 µm in diameter within 4 weeks. Moreover, the spherical organoids derived from HEKs gradually progressed into a lumen‐containing morphology with visible lumen surrounded by single‐ or multiple‐layered epithelial cells after 2 to 3 weeks’ culture in SGM supplemented with ISO (Figure 4b–e). We next investigated the cellular identity of the iSwGOs in culture. Immunofluorescence analyses revealed that the iSwGOs contained cells corresponding to ductal cells, luminal cells, and myoepithelial cells, which were individually characterized by expression of CK5 and CK10 (37.88 ± 5.12%, Figure 4f,g), AQP5 and CK18 (33.32 ± 3.9%, Figure 4f,h), and CK5 and *α*‐SMA (35.12 ± 4.49%, Figure 4f,i). We further used FACS to isolate the myoepithelial population from iSwGCs (Figure S13a, b, Supporting Information) on the basis of CD49f and CD29 expression.^[^ ^2^, ^41^ ^]^ The FACS‐purified CD49f^hi^CD29^hi^ ‐expressing cells were enriched in myoepithelial markers (Figure S13c, Supporting Information) and showed a markedly increased organoid growth capacity as compared to CD49f^lo^CD29^lo^ cells (Figure S13d, Supporting Information). It is of note that luminal‐specific CK18 and CK19 expression was restricted to the central lumen (inner layers), with *α*‐SMA‐expressing cells surrounding at the outer layer in the iSwGOs derived from CD49f^hi^CD29^hi^ cells (Figure 4j and Figure S13e, Supporting Information), which was the same as that found in native SwGs. We also established iSwGOs from the reprogrammed HaCaTs with the same method (Figure S12a–f, Supporting Information). Collectively, these results demonstrate that iSwGCs could develop into iSwGOs via long‐term 3D culture. The resulting iSwGOs retained the typical cyto‐organization and histological features closely resembling the native SwG bulk in vivo.

We next explored the functional properties of iSwGOs. First, we observed the presence of intracellular Ca^2+^ activity in the epithelial cells of iSwGOs after exposure to a Ca^2+^ fluorescent indicator (Figure 4k). Second, given that the established iSwGOs exhibited cellular diversity, polarization, and cystic structure resembling their in vivo counterparts, we sought to test whether these iSwGOs would be able to self‐assemble into macroscopic tubular structures in response to FGF gradients^[^ ^42^ ^]^ in vitro, which is considered necessary for the in vivo functionalization of SwGs.^[^ ^2^, ^6^ ^]^ Given the advantages of microfluidic platforms in study of gradient‐induced cell behaviors, iSwGOs (≈30 µm in diameter) derived from GFP‐labeled HEK‐EDA cells were mixed in Matrigel at a 1:3 ratio and plated into the microfluidic culture chambers (≈30 organoids per chamber; chamber area < 4 mm^2^). Concentration gradients of bFGF were created in microfluidic devices based on the osmotic pump due to the concentration difference between microchip channels (Figure S14a,b, Supporting Information). As expected, the elongation of iSwGOs with a bipolar shape along the gradient direction was observed 8 days after exposure to bFGF gradients (Figure S14c, Supporting Information). After 18 days’ culture, the iSwGOs maintained in microfluidic chambers formed typical tubular structures with hollow lumens that were lined with a simple epithelium (Figure 4l and Figure S14d, Supporting Information). Immunofluorescence analysis further showed that the epithelial cells in the tube were immunopositive for SwG ductal markers, luminal markers and myoepithelial markers (Figure 4l), which suggests that the iSwGOs were capable of responding to the chemotaxis of extracellular signaling molecules to form tubular structures in vitro.

Taken together, these results demonstrate that iSwGCs could self‐assemble into organoids in a special 3D culture system, which showed characteristic SwG properties and were able to respond to the extracellular biological cues for further development toward functional SwGs.

### 2.5 Transplanting iSwGOs In Vivo Reconstituted Damaged Skin with Fully Restored SwG Functions

**Figure 5:**
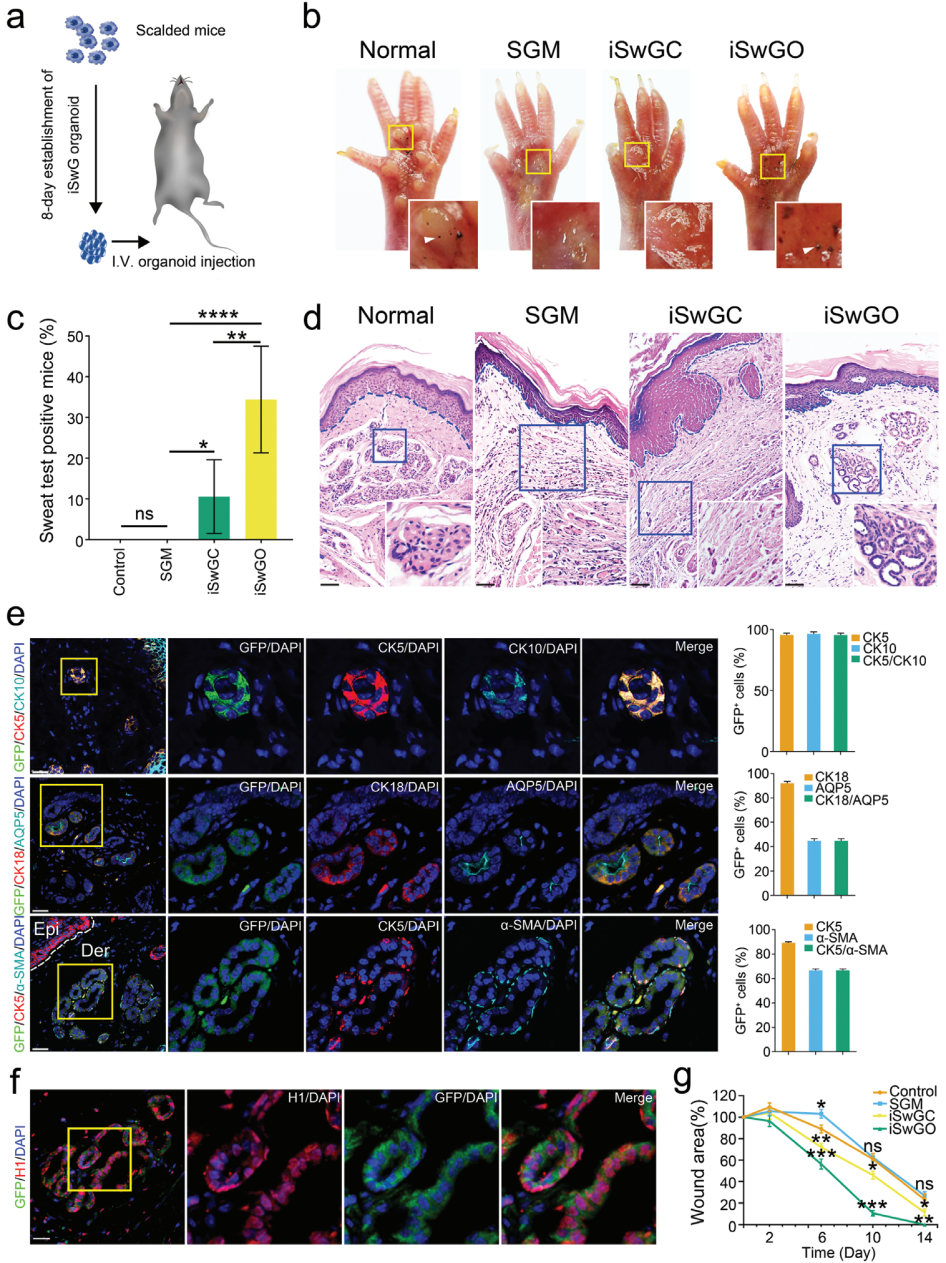
Engraftment of iSwGOs functionally repopulated damaged skin. a) Schematic diagram representing the experimental procedure; b) Starch–iodine sweat tests on paw skin of thermal‐injured mice showed that only paws of iSwGO‐treated mice responded by displaying indigo‐black dots at day 21 after transplantation; c) Starch–iodine assessment of newly formed SwGs in the defect regions of thermally injured mice at day 30 after SGM, iSwGC and iSwGO treatment. The number of mice positive for starch‐iodine reaction increased in a time‐dependent manner. After 30 days treatment with iSwGOs, 34.4 ± 13.0% of the recipient mice (*n* = 60 per group) exhibited sweat production as compared with those treated with iSwGCs (13.3 ± 10.6%, *n* = 60), SGM (0, *n* = 60) and vehicle control (0, *n* = 60). Data are mean ± SD of 6 independent experiments; d) H&E staining was conducted to visualize SGM‐, iSwGC‐, iSwGO‐treated wounds at day 21 post‐injury. Emerging glandular structures were seen in the dermis of iSwGO‐treated mice. Dotted line represents the ridges of epidermis where the sweat pores open. Note that the rete ridges in iSwGO‐engrafted paw skin were elongated and intertwined with underlying dermal tissues comparable to those derived from normal skin. Scale bar = 100 µm; e) Immunofluorescence and quantification of SwG marker expression in nascent glands at day 21 after iSwGO transplantation (left). EDA‐GFP^+^ cells were seen in the newly generated glandular structures. Newly emerged glands showed typical SwG morphological features and expressed SwG specific markers, for example, ductal markers CK5 and CK10, luminal markers AQP5, CK18, and myoepithelial markers CK5 and *α*‐SMA. Scale bar = 25 µm. Quantification (right) involved > 150 cells from 3 independent experiments, and GFP^+^ cells within the duct, luminal, and myoepithelial domain were measured, respectively; f) Immunofluorescence of human‐specific histone protein expression in nascent SwGs at day 21 after iSwGO transplantation. Scale bar = 25 µm; g) Wound healing curves for quantification of the wound coverage at different times in SGM‐, iSwGC‐, iSwGO‐treated mice (*n* = 15, 3 independent experiments). Mice treated with DMEM/F‐12 were vehicle controls. Data are mean ± SD; * *p* < 0.05, ** *p* < 0.01, *** *p* < 0.001.

To assess the tissue reconstitution potential of HEK‐derived iSwGOs in vivo, we created deep partial‐thickness scald burns in foot pads of mice, which produced a deep dermal injury with complete destruction of the SwGs. GFP‐labeled organoids, iSwGCs, or SGM were injected subcutaneously into the hind paws of scalded mouse pads (**Figure** **5a**). Strikingly, on starch–iodine sweat tests, only paws of iSwGO‐treated mice showed indigo‐black dots at day 21 after iSwGO transplantation (Figure 5b), reflecting a functional restoration of SwGs in the thermally injured mice. The number of mice positive for starch–iodine reaction increased time‐dependently, and a mean of 34.4 ± 13.0% of recipient mice exhibited sweat production at day 30 after treatment with iSwGOs (*n* = 60), which was significantly higher than that for mice treated with iSwGCs (13.3 ± 10.6%, *n* = 60) or SGM (0, *n* = 60, Figure 5c).

To determine the contribution of engineered organoids to SwG regeneration, we analyzed the grafts of iSwGOs, and nascent ductal and glandular structures were seen in the dermis of recipient mice at 21 days after transplantation (Figure 5d). Tissue sections of GFP^+^ areas further showed that GFP^+^ cells and their progeny were integrated into the proliferative epidermis or formed glandular structures with characteristic SwG features (Figure 5e). Of note, immunostaining of human‐specific histone protein further confirmed the human origin of GFP^+^ glandular structures in the mouse paw skin (Figure 5f). iSwGO engraftment also promoted wound closure in vivo. At 10 days after treatment, macroscopic measurements and morphometric analysis showed a significant difference in the mean wound area in mice treated with iSwGOs (mean 10.7 ± 2.1% of initial size), iSwGCs (46 ± 2.9%) and SGM (62.7 ± 2.9%) (Figure 5g and Figure S15a, Supporting Information). At day 14 after transplantation, the wounds in iSwGO‐treated mice were covered completely with proliferative epidermis, whereas a mean of 88.7 ± 2.6% of wounds treated with iSwGCs were covered by neo‐epidermis. In contrast, only a mean of 73 ± 2.9% of the wounds were covered in SGM groups at the same time (Figure 5g). Collectively, our data demonstrate that iSwGOs derived from reprogrammed HEKs successfully repopulated damage skin with fully restored SwGs in vivo.

## 3 Discussion

One key question in treating extensive skin defects is how to obtain functional cells for large‐scale application cost‐effectively. Lack of donor tissues and low ex vivo expansion capacity of primary SwG cells hamper the transplantation of autologous or xenogenous SwG‐derived cells for therapy in the clinic. Also, wound‐resident SwG progenitor cells are not sufficient to orchestrate the healing process for promotion of de novo SwG morphogenesis. Our study demonstrated a feasible and promising approach for generating patient‐specific SwG cells and provided a desirable cell resource for functional treatment of extensive skin defects. In the present study, we first showed that reprogrammed SwG cells can be generated from HEKs transduced with a single factor, EDA, under defined SwG induction conditions. Within the context of SwG reprogramming, EDA expression primed an intrinsic possibility for driving the fate conversion of HEKs, whereas generation of iSwGCs highly depended on the addition of SwG conditional medium. Specifically, activation of *β*_2_‐AR signaling efficiently improved the SwG cell conversion from stably transduced human keratinocytes by priming cellular stemness and gland development‐associated gene expression programs within the initial HEKs. Systemic transcriptome profiling and function enrichment analysis showed consistently that the gene expression patterns of related GO terms in iSwGCs were similar to those of native SwG cells. Thus, our two‐step reprogramming strategy could facilitate the SwG reprogramming of HEKs transduced with EDA alone, and the resulting iSwGCs acquired characteristic properties of functional SwG cells.

Another important feature on the applied side of our work is the generation of gene‐engineering functional organoids for in vivo replacement of damaged SwGs. Human SwG organoids derived from SwG epithelial cells contributed to the regeneration of epidermis and SwGs in vivo.^[^ ^22^ ^]^ In patients with large skin defects, the pathological microenvironment within the wound bed has been a major roadblock for functional reconstruction of damaged skin. Accordingly, methods combining organoid cultures with reprogramming technology hold great promise for generating a sufficient amount of tissue‐relevant organoids for restoring vital SwG functions during wound healing. They also alleviate the pathological conditions enabling a permissive environment in the wound bed. In our study, we established functional human SwG organoids from iSwGCs. After single‐cell plating, the 3D culture system mimicking the paracrine environment of in vivo SwGs led to rapid expansion of the engineered organoids. These organoids recapitulated critical aspects of SwG biology. An important feature is that the iSwGOs were able to self‐assemble into tubular structures along a bFGF gradient in vitro. These tubular structures retained a SwG epithelial identity and morphologically resembled immature SwGs, so spatio‐temporal biological cues from the extracellular microenvironment may play crucial roles in guiding and extending the morphogenetic process of SwGs in vitro and in vivo. Another key feature of the iSwGOs is their ability to repopulate damaged skin structurally and functionally. GFP‐labeled cells were robustly observed in the regenerative epidermis and nascent glands. Importantly, the emerging SwGs exhibited structural and phenotypical features characteristic of native SwGs, consisting of both duct and secretory domains and showing responsiveness to sweat test assays when engrafted onto paw skin of thermo‐injured mice. Of note, iSwGOs generated epidermal coverage more rapidly and enabled de novo SwG morphogenesis efficiently as compared with iSwGCs. Collectively, the iSwGOs derived from reprogrammed epidermal keratinocytes could be a tool to study SwG development and provide primes for in vivo tissue replacement therapy.

In conclusion, we demonstrate a practical and feasible strategy for achieving functional SwG regeneration by using engineering human SwG organoids in vivo. This strategy enables the efficient conversion of HEKs into SwG lineages and permits the generation of desired functional organoids for in vivo treatment. The generation of iSwGOs with reprogramming approaches highlights a way to obtain reproducible and functional human organoids in a personalized fashion, treating or replacing injured skin tissues structurally and functionally.

## 4 Experimental Section

### Tissue Samples

All specimens from human sources were obtained with the informed consent of patients and institutional approval (Clinical Research Ethics Committee of General Hospital of PLA, project No. S2020‐460‐01). Foreskin tissue samples were obtained from healthy youth (age 15–19 years old) undergoing circumcision. Normal skin tissue was obtained after breast plastic surgery. Skin tissues from fetuses at 23 to 30 weeks' gestation were obtained from the tissue bank of our laboratory. The present studies were approved by the Clinical Research Ethics Committee of General Hospital of PLA (Beijing) and written informed consent was obtained from all individuals prior to obtaining samples.

### Cell Culture and Treatment

Primary HEKs were isolated from male foreskin specimens. Briefly, skin tissues were cut into pieces and incubated with 0.5% dispase II (Sigma‐Aldrich) at 4 °C for 12–18 h. The epidermis was then separated from underlying dermal connective tissue and digested with 0.25% trypsinase for 20 min at 37 °C. After washing for three times with phosphate buffered saline (PBS), the single keratinocytes were resuspended and seeded at 1 × 10^6^ cells cm^−2^ on collagen IV‐coated culture flasks (Type IV collagen was from Sigma‐Aldrich). After 20‐min incubation at 37 °C, attached cells were maintained in Epilife medium (Thermo Fisher Scientific) supplemented with 1% human keratinocyte growth supplement (Thermo Fisher Scientific) and 1% penicillin/streptomycin (Solarbio, Beijing).

Human immortalized keratinocyte (HaCaT) cells were purchased from the China Infrastruture of Cell Line Resources (3111C0001CCC000373; Beijing), and cultured in basic Epilife medium (Thermo Fisher Scientific) supplemented with 0.06 mm Ca^2+^ and 1% Epilife defined growth supplement (Thermo Fisher Scientific). HEK‐EDA cells were cultured in SwG‐specific medium (SGM), and HaCaT‐EDA cells were cultured in SGM with 5 µg mL^−1^ Dox (Sigma). MGC‐803 cells were maintained in RPMI 1640 (Gibco) supplemented with 10% fetal bovine serum (FBS; Gibco). For simulation of *β*_2_‐adrenergic receptor (*β*_2_‐AR), cells were starved overnight and treated with *β*_2_‐AR agonist ISO (5 µm, Sigma‐Aldrich) for 48 h. For *β*_2_‐AR antagonist treatment, starved cells were pretreated with 10 µm ICI‐118551 (Sigma‐Aldrich), a selective *β*_2_‐AR antagonist, for at least 3 h before ISO stimulation.

### Sample Preparation and Staining

For immunofluorescence staining, cells were fixed in 4% paraformaldehyde (Solarbio) at room temperature for 30 min and permeabilized in 0.2% Triton ×100 in PBS for 10 min. Paraformaldehyde‐fixed tissue was rinsed in PBS, then permeabilized. Organoids in a microfluidic chamber were fixed for 48 h and blocked with PBS containing 0.25% Triton X‐100 and 5% normal goat serum (Solarbio) at room temperature for 12 h. Then cell and tissue samples were probed with primary antibodies at 4 °C overnight and secondary antibodies at room temperature for 2 h. The primary antibodies used in this study were rabbit anti‐CK5 (ab52635, Abcam, 1:500), mouse anti‐CK5 (ab181491, Abcam, 1:800), mouse anti‐HA (2367s, CST, 1:100), rabbit anti‐Histone (ab125027, Abcam, 1:100), rabbit anti‐EDA (ab125233, Abcam, 1:200), mouse anti‐CK10 (ab9025, Abcam, 1:200), rabbit anti‐CK19 (ab52625, Abcam, 1:500), mouse anti‐CK19 (ab7754, Abcam, 1:500), mouse anti‐CK18 (4548s, CST, 1:800), mouse anti‐CK14 (ab9220, Abcam, 1:100), mouse anti‐*α*‐SMA (48938s, CST, 1:800), rabbit anti‐AQP5 (ab92320, Abcam, 1:50), rabbit anti‐NKA (23565s, CST, 1:100). rabbit anti‐*β*_2_‐AR (ab182136, Abcam, 1:100). The following secondary antibodies were used: goat anti‐mouse IgG H&L (Alexa Fluor 488) (ab150113, Abcam, 1:200), goat anti‐mouse IgG H&L (Alexa Fluor 555) (ab150114, Abcam, 1:200), goat anti‐rabbit IgG H&L (Alexa Fluor 555) (ab150078, Abcam, 1:200), goat anti‐mouse IgG H&L (Alexa Fluor 647) (ab150115, Abcam, 1:200), goat anti‐rabbit IgG H&L (Alexa Fluor 647) (ab150079, Abcam, 1:200). Organoids in the microfluidic chamber were incubated with primary and secondary antibodies for 48 and 24 h, respectively. Imaging for immunofluorescence involved using a Leica fluorescence microscope.

For immunohistochemistry staining, antigen retrieval of samples was carried out in 10 mm citric acid buffer (pH 6.0) for 15 min, and 0.3% H_2_O_2_ was added to block endogenous peroxidase activity. Then slides were incubated with rabbit anti‐*β*_2_‐AR (ab182136, Abcam, 1:200) overnight at 4 °C. Antibody binding was detected by using a streptavidin‐biotin‐peroxidase immunohistochemical system (SP‐9000, ZSGB‐BIO), and color development was detected by DAB staining (ZLI‐9017, ZSGB‐BIO). The slides were counterstained with hematoxylin.

### Flow Cytometry Sorting and Analysis

Digested cells were suspended and washed with PBS containing 5% FBS to make a single‐cell suspension. GFP‐positive populations were enriched by using a FACSCalibur flow cytometry system (BD Bioscience) with FlowJo software. Flow cytometry assays were performed by using a BD FACSCanto I flow cytometer (BD Bioscience). Antibodies used were: APC rabbit anti‐CK5 (ab224984, Abcam, 1:5000), PE mouse anti‐CK10 (NBP2‐34752PE, Novus, 1: 500), PE mouse anti‐*α*‐SMA (NBP2‐34522PE, Novus, 1:100), PE rabbit Anti‐CK18 (ab210410, Abcam, 1:200), Alexa Fluor 647 rabbit anti‐AQP5 (ab215225, Abcam, 1:100), APC rabbit anti‐CD49f (313 615, Biolegend, 1:1000), and PE mouse anti‐CD29 (303 003, Biolegend, 1:1000). Gating was performed on single‐stained controls and unstained controls.

### Molecular Cloning and Generation of iSwGCs

The sequence of sgRNA targeting to EDA transcriptional start site (F:TCGGCCCGCCGAGGGAATGA, R:TCATTCCCTCGGCGGGCCGA) and the plasmid encoding dCas9‐E (pHAGE‐TRE‐dCas9‐VP64) were obtained from previous research in our lab.^[^ ^4^ ^]^ The sgRNA expression plasmid (pLV‐H1‐sgRNA‐GFP) was from GenePharma (Suzhou, China). The plasmid overexpressing EDA (pLV‐hef1a‐EDA‐GFP) was from SyngenTech (Beijing). HEK‐293FT cells obtained from our lab and were maintained in DMEM supplemented with 10% FBS. Lentiviral packaging plasmids were co‐transfected with sgRNA, dCas9‐E or pLV‐hef1a‐EDA‐GFP plasmids by using Lipofectamine 2000 (Invitrogen) according to the manufacturer's instructions. GFP‐positive keratinocytes were enriched by flow cytometry sorting, and double transfected cells were screened by using 400 µg mL^−1^ G418.

### qPCR

Total RNA was extracted by using the RNAgents Total RNA Isolation System (Promega) with DNase I (Invitrogen) treatment. cDNA synthesis involved using the PrimeScript RT reagent kit (TaKaRa). qPCR involved using the SYBR Green I on GoTaq qPCR Detection System (Promega) according to the manufacturer's instructions. Quantification of target genes was normalized to the expression of GAPDH. The primer information is in Table S1, Supporting Information.

### RNA‐Sequencing and Statistics

Construction of an RNA‐seq library, Illumina sequencing, and bioinformatics analysis were performed at Novogene Bioinformatics (Beijing). Total RNA was isolated from HEKs with or without ISO administration by using the RNeasyMini kit (QIAGEN). After purification, RNA sequencing libraries were prepared by using the NEBNext Ultra RNA Library Prep Kit for Illumina (New England Biolabs) following the manufacturer's recommendations and sequenced on an Illumina/Hiseq‐2500 platform to generate the 125/150‐bp paired‐end reads. For each sample, three independent biological replicates were used for RNA‐seq. Gene expression was normalized by DESeq2 (1.16.1), and the low expression genes with total counts across all samples < 1 were excluded in our data. Analysis of RNA‐seq data involved using the cluster Profiler R package, and GO terms with corrected P < 0.05 were considered significant.

### Western Blot Analysis

Total protein was isolated from cells by using a RIPA buffer (Sigma‐Aldrich). Subsequently, whole cell lysates were prepared, separated by SDS‐PAGE, and transferred to PVDF membranes (GE Healthcare). Blots were probed with the following primary antibodies overnight at 4 °C: rabbit anti‐SOX9 (ab185966, Abcam, 1:1000), rabbit anti‐LGR5 (ab75850, Abcam, 1:1000), rabbit anti‐OCT4 (ab19857, Abcam, 1:1000), mouse anti‐CK18 (4548s, CST, 1:2000), rabbit anti‐EDA (ab125233, Abcam, 1:500), mouse anti‐CEA (2383s, CST, 1:1000), rabbit anti‐BMP5 (ab38565, Abcam, 1:1000), rabbit anti‐CK19 (ab52625, Abcam, 1:50 000), rabbit anti‐CK5 (ab52635, 1:10 000), rabbit anti‐c‐MYC (ab32072, Abcam, 1:1000), rabbit anti‐*α*‐SMA (19245s, CST, 1:1000), rabbit anti‐AQP5 (ab92320, Abcam, 1:1000), rabbit anti‐*β*_2_‐AR (ab182136, Abcam, 1:1000), rabbit anti‐HA (ab9110, Abcam, 1:4000), and rabbit anti‐GAPDH (2118s, CST, 1:1000). The blots were then washed and incubated with horseradish peroxidase‐conjugated secondary antibodies (sc‐2005, sc‐2004, Santa Cruz, 1:1000). Bands were detected by enhanced chemiluminescence (Pierce).

### 3D and Microfluidic Culture of iSwGOs

For iSwGO 3D culture, cells were resuspended at the required density, embedded in Matrigel and cultured in SGM medium [advanced DMEM/F‐12, supplemented with 2 mm GlutaMax, 10 mm HEPES, B‐27 supplement (1× ), 1 mm N‐acetylcysteine, 10 mm nicotinamide (Sigma‐Aldrich), and 1% penicillin/streptomycin] containing 50 ng mL^−1^ human recombinant EGF (Sigma‐Aldrich), 20 ng mL^−1^ human recombinant fibroblast growth factor (bFGF; Sigma‐Aldrich), and 5 µm ISO with or without 20 ng mL^−1^ human recombinant BMP4 protein and 10 µm CHIR99021, a medium modified from those previously used.^[^ ^22^ ^]^ The presence of BMP4 and CHIR99021 improved the efficiency for the formation of CK5^+^/*α*‐SMA^+^ iSwGOs during 3D cultures.

The microfluidic culture system was provided by Prof. Yue (Shanghai University). After sterilization, matrigel–organoid suspensions (50 organoids/matrigel drop) were loaded into a microfluidic chamber via inlet port ① (Figure S14b, Supporting Information) using a micropipette. The microchip channels were infused with modified SGM through inlets ②, ④ and ③, ⑤ with a 1000‐µL pipette (Figure S14b, Supporting Information). A concentration gradient was generated in the culture chamber after medium containing different concentrations of bFGF entered the channels ②–④ and ③–⑤. Microfluidic cultures were maintained in 37 °C and the medium was changed 3–4 times a week.

### Animals and Transplantation

The animal experiment was performed according to the protocols approved by the Ethics Committee at the Fourth Medical Center of PLA General Hospital and in accordance with Institutional Animal Care and Use Committee guidelines (approval No. 2019‐X‐15‐50). For establishing a mouse burn model, athymic BALB/c nude mice (female, 8 weeks old) were purchased from HuaFuKang Bioscience (Beijing). The mice were anesthetized with pentobarbital (100 mg/kg) and second‐degree burn was administered to the hind paws of mice for 5 s to destroy SwGs in the dermis. At 3 and 5 days after thermal injury, cells (5 × 10^5^ cells in 50 µL SGM medium) and organoids (5 × 10^4^ in 50 µL Matrigel and SGM mixture) were collected and injected into the paw pads of recipient mice. Simultaneously, 50 µL SGM was intradermally injected in the scalded paw pads, and mice injected with 50 µL DMEM/F‐12 were vehicle controls. After the injection, mice were monitored daily and sacrificed at indicated days.

### Calcium Activity Analysis

Intracellular Ca^2+^ measurement was performed by using Calcium Orange Indicators (ThermoFisher Scientific) according to the manufacturer's instructions. Live cells and organoids were loaded directly with AM ester calcium indicators for 10 min at room temperature. Then, fluorescence signals were measured by using fluorescence microscopy and flow cytometry upon addition of 50 µm ACh.

### Sweat Test

The paw pads were first painted with 2% (w/v) iodine/ethanol solution and then starch. After drying, 50 µL of 100 µm ACh (Sigma) was injected subcutaneously into the paws of mice. The sweat test was performed weekly in the first 2 weeks and every 3 days in the next 2 weeks to evaluate function of nascent SwGs.

### Electron Microscopy

Cells were harvested and fixed with 2.5% glutaraldehyde in phosphate buffer at room temperature for > 2 h. Cell samples were first dehydrated by a graded series of ethanol for 15 min at each step and transferred to absolute acetone for 20 min. Resin was then used for sample infiltrating and embedding. Samples were prepared as ultrathin sections and observed with the Hitachi TEM system.

### Statistical Analysis

Data are presented as mean ± standard deviation (SD). The data conformed to the normal distribution, and the variance was homogeneous. Statistical analysis was performed with SPSS 20.0. Comparisons between 2 groups were analyzed by Dunnett's *t*‐test, and between more than 2 groups by one‐way ANOVA. *p* < 0.05 was considered statistically significant.

## Author Contributions

X.S., J.X., R.C., and Z.G. contributed equally to this work. X.Y.S., L. D., and X.B.F. conceived ideas and designed the experiments. X.Y.S., J.B.X., and R.K.C. performed the experiments and analyzed the results. L.T.W., Y.Q.L., S.F.J, H.T.C, Y.L., and C.P.Z. analyzed the results. L.D. reviewed & edited the manuscript. P.L. contributed to high throughput screening. Z.J.G. provided technical assistance. X.Y.S., L.D., and X.B.F. wrote the manuscript. T.Y. designed and fabricated microfluid chips, and contributed to manuscript editing.

## Conflict of Interest

The authors declare no conflict of interest.

## Supporting information

The data that supports the findings of this study are available in the supporting information of this article.

## Data Availability

The data that supports the findings of this study are available in the supplementary material of this article
