# Supplementary material for: Sweat Gland Organoids Originating from Reprogrammed Epidermal Keratinocytes Functionally Recapitulated Damaged Skin
Source: Adv Sci (Weinh). 2021 Sep 26;8(22):2103079. doi: 10.1002/advs.202103079 (PMC8596119; doi:10.1002/advs.202103079)
Supplement: Supplementary file 1 — The data that supports the findings of this study are available in the supporting information of this article. [file ADVS-8-2103079-s001.pdf]

© 2021 Wiley-VCH GmbH

ADVANCED SCIENCE
Open Access

Supporting Information

for *Adv. Sci.*, DOI: 10.1002/advs.202103079

Sweat gland organoids originating from reprogrammed epidermal keratinocytes functionally recapitulated damaged skin

Xiaoyan Sun[1,#], Jiangbing Xiang[1,2, #], Runkai Chen[1,3,#], Zhijun Geng[1,#], Lintao Wang[4], Yiqiong Liu[1], Shuaifei Ji[1], Huating Chen[1], Yan Li[1], Cuiping Zhang[1], Peng Liu[5], Tao Yue[6,7,], Lei Dong[4,*], Xiaobing Fu[1,*]*

Supplementary Materials for

Sweat gland organoids originating from reprogrammed epidermal keratinocytes functionally recapitulated damaged skin

Xiaoyan Sun[1, #], Jiangbing Xiang[1, 2, #], Runkai Chen[1, 3, #], Zhijun Geng[1, #], Lintao Wang[4], Yiqiong Liu[1], Shuaifei Ji[1], Huating Chen[1], Yan Li[1], Cuiping Zhang[1], Peng Liu[5], Tao Yue[6, 7, *], Lei Dong[4, *], Xiaobing Fu[1, *]

[1] Research Center for Tissue Repair and Regeneration affiliated to the Medical Innovation Research Department and 4th Medical Center, PLA General Hospital and PLA Medical College; PLA Key Laboratory of Tissue Repair and Regenerative Medicine and Beijing Key Research Laboratory of Skin Injury, Repair and Regeneration; Research Unit of Trauma Care, Tissue Repair and Regeneration, Chinese Academy of Medical Sciences, 2019RU051, Beijing 100048, P. R. China

[2] Bioengineering College of Chongqing University, Chongqing 400044, P. R. China.

[3] Department of General Surgery, Chinese PLA General Hospital, 28 Fu Xing Road, Beijing 100853, P. R. China

[4] State Key Laboratory of Pharmaceutical Biotechnology, School of Life Sciences, Nanjing University, Nanjing, Jiangsu 210023, China.

[5] Department of Biomedical Engineering, School of Medicine, Tsinghua University, Haidian District, Beijing, 100084, China.

[6] School of Mechatronic Engineering and Automation, Shanghai University, Shanghai 200444, China

[7] Shanghai Institute of Intelligent Science and Technology, Tongji University, Shanghai 200092, China

***Correspondence:** Xiaobing Fu, Research Center for Tissue Repair and Regeneration affiliated to the Medical Innovation Research Department and 4th Medical Center, PLA General Hospital and PLA Medical College, 28 Fu Xing Road, Beijing 100853. Tel. 0086-10-66936345; Fax: 0086-10-66936345; e-mail: fuxiaobing@vip.sina.com

Correspondence may also be addressed to: Lei Dong, State Key Laboratory of Pharmaceutical Biotechnology, School of Life Sciences, Nanjing University, Nanjing, Jiangsu 210023, China; e-mail: leidong@nju.edu.cn

Tao Yue, School of Mechatronic Engineering and Automation, Shanghai University, Shanghai 200444, China; e-mail: tao_yue@shu.edu.cn

#Co-first author: the authors contributed equally to this paper.

The PDF file includes:

Figure S1 EDA alone was insufficient to achieve SwG conversion.

Figure S2 Reprogramming of human epidermal keratinocytes into SwG cells by overexpression of EDA combined with SGM.

Figure S3 Enhanced stemness induced cell fate switch of HEK-EDA cells into SwG lineages.

Figure S4 Beta2-AR was expressed in the secretory domain of adult SwGs.

Figure S5 Gene Ontology functional analysis and KEGG pathway enrichment analysis of differentially expressed genes in HEKs.

Figure S6 Beta2-AR activation upregulated stemness-associated gene expression in HaCaTs.

Figure S7 Beta2-AR activation upregulated specific SwG gene expression in HEKs.

Figure S8 Activation of β_2-ARs upregulated of SwG-specific genes in iSwGCs.

Figure S9 Generation of iSwGCs from HaCaT keratinocytes via CRISPR-mediated EDA activation together with optimized induction conditions.

Figure S10 HaCaT-derived iSwGCs expressed SwG functional markers.

Figure S11 Heatmap of RNA-seq data displaying the expression of the indicated genes during iSwGC conversion.

Figure S12 Generation of iSwGOs from reprogrammed HaCaTs.

Figure S13 Generation of organoids from iSwGCs with myoepithelial identity.

Figure S14 Self-organization of iSwGOs into tubular structures directed by bFGF gradients.

Figure S15 iSwGO transplantation promoted in-vivo skin wound healing.

Other Supplementary Material for this manuscript includes the following:

1. Supplementary methods
2. Table S1 Primer sequences used in the study

Supplementary Materials

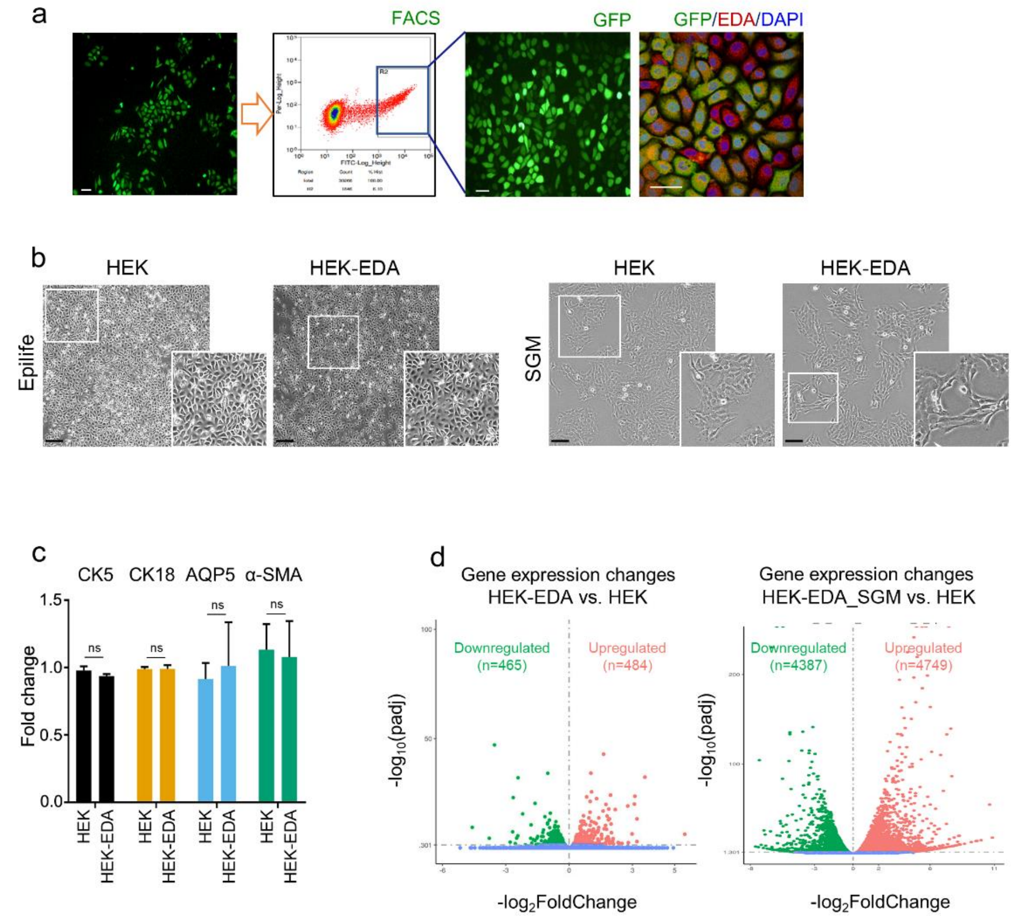

Figure S1 EDA alone was insufficient to achieve SwG conversion. (a) Schematic of the protocol used for generating EDA-overexpressing HEKs. After transfection of HEKs with pLV-hef1a-EDA-GFP, the GFP-positive population was FACS sorted and the expression of EDA was validated by immunofluorescence. Scale bar = 50 μm; (b) Phase-contrast images of HEK-EDA cells cultured in Epilife or SGM conditions. Scale bar = 100 μm. Insets, higher magnification of the boxed areas; (c) qPCR array showing transcriptional expression of SwG genes CK5, CK18, AQP5, α-SMA in HEKs with or without EDA transduction. n = 3 for all measurements. Data are mean ± SD and were analyzed by two-tailed t-tests. ns, not significant; (d) Volcano plot showing all transcripts that are log_2-fold changed in HEK-EDA cells, SGM-treated HEK-EDA cells compared to HEKs. Every dot represents a gene. n = 3 biological replicates.

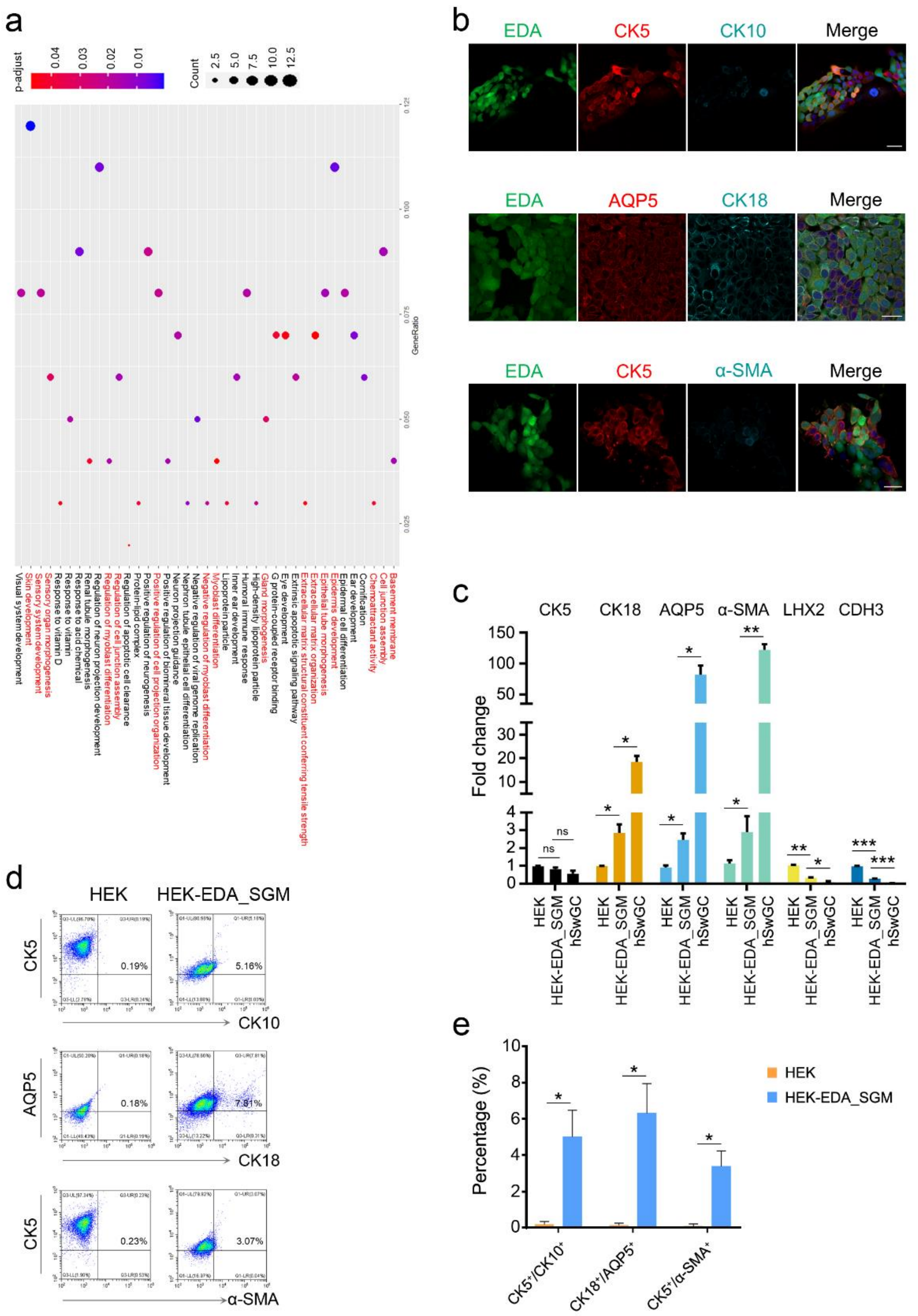

Figure S2 Reprogramming of human epidermal keratinocytes into SwG cells by overexpression of EDA combined with SGM. (a) Gene set enrichment analysis of HEK-EDA cells versus HEK samples showing Gene Ontology terms related to SwG development among the top enriched; (b) Immunofluorescence of SGM-treated HEK-EDA cells for ductal markers, luminal markers, and myoepithelial markers. Scale bar = 50 μm; (c) qPCR analysis showing transcriptional expres-

sion of SwG (CK5, CK18, AQP5, α-SMA)- and hair follicle (LHX2, CDH3)-specific genes in HEKs and SGM-treated HEK-EDA cells. Primarily isolated sweat glands cells from human skin sample were positive controls. The genes showing significant changes in qPCR analysis are presented (n = 3); (d, e) FACS analysis showing phenotypical changes of HEKs and SGM-treated HEK-EDA cells. n = 3, “n” represents the number of biological replicates. Data are mean ± SD and were analyzed by two-tailed t-tests, *$P < 0.05$, **$P < 0.01$, ***$P < 0.001$. ns, not significant.

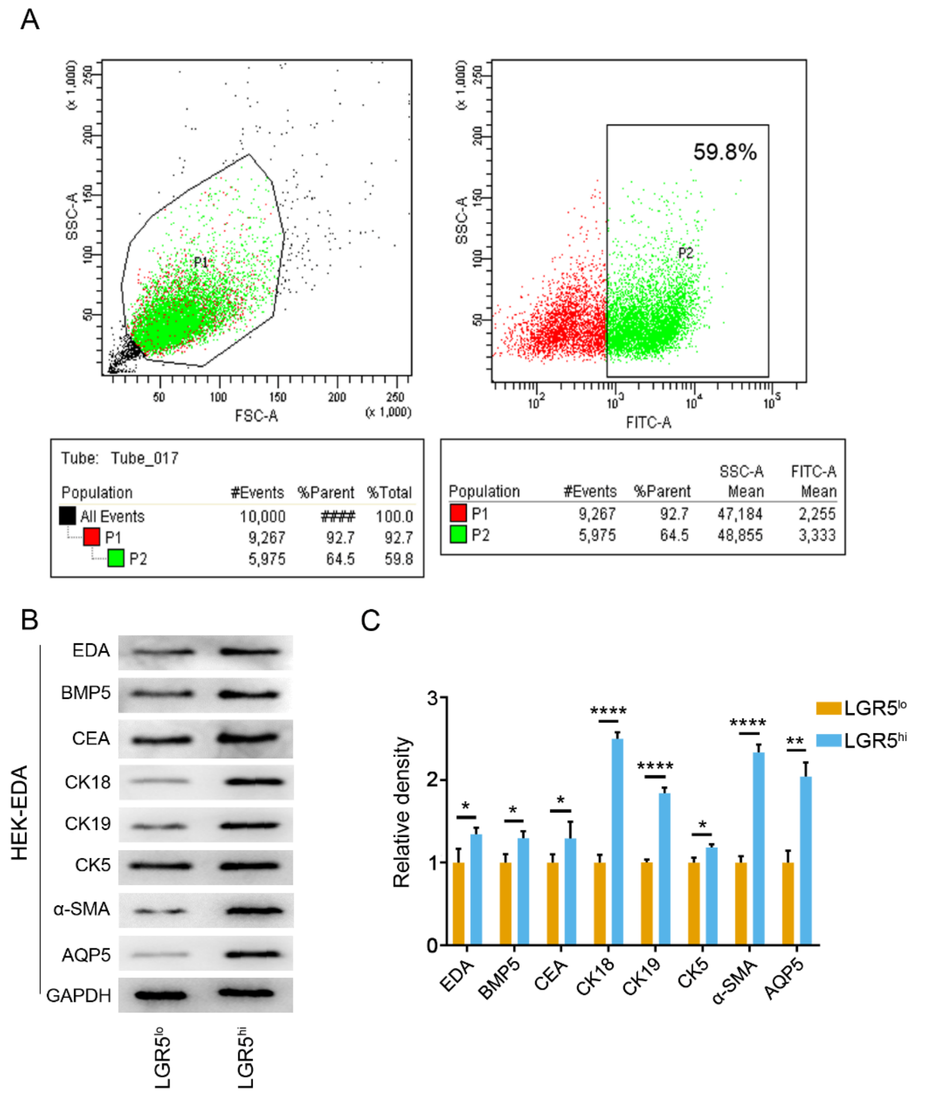

Figure S3 Enhanced stemness induced cell fate switch of HEK-EDA cells into SwG lineages. (a) Representative image showed gating for viable cells (P1 gate) from non-viable cells and debris (left), and gating P1 fraction for LGR5 fluorescence (right). P2 gate represented the LGR5^{hi} fraction in HEKs; (b) Following FACS sorting based on LGR5 expression, LGR5^{hi} and LGR5^{lo} cells were transduced with EDA and cultured in SGM. Western blot analysis of EDA overexpression in LGR5^{hi} cells and protein levels of BMP5, CEA, CK18, CK19, CK5, α-SMA and AQP5 expression as compared with LGR5^{lo} cells; (c) Quantitative western blot analysis of protein expression for EDA, BMP5, CEA, CK18, CK19, CK5, α-SMA and AQP5. n = 3 for the measurements, "n" represents the number of biological replicates. *$P < 0.05$, **$P < 0.01$, ****$P < 0.0001$.

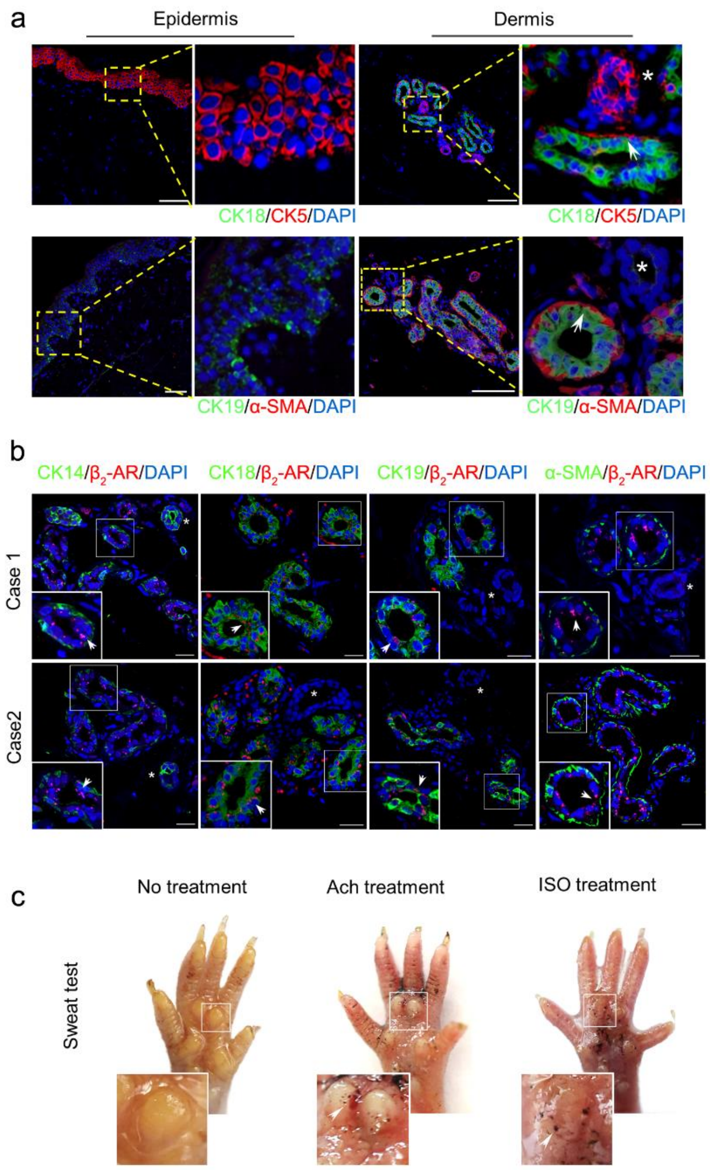

Figure S4 Beta2-AR was expressed in the secretory domain of adult SwGs. (a) Immunofluorescent staining for CK5, CK18, CK19 and α-SMA in human epidermis and SwGs. CK5 labeling cells (red, up row) located in the basal and suprabasal layers of epidermis (left) and the ductal (star) and myoepithelial cells (arrow) of SwGs (right). CK19 (green, bottom row) were mainly detected in the basal layer of epidermis. In SwGs, both CK18 (green, up row) and CK19 (green, bottom row) were expressed in the inner layer of luminal cells, while CK5 (arrow, up row) or α-SMA (red, bottom row) marking myoepithelial cells were detectable in the outer layer of the secretory coils. Scale bars = 50 μm; (b) Representative immunofluorescence images showing the co-labelling of

β_2-AR with CK14, CK18, CK19 and α-SMA in human mature SwGs. White arrows show the inner luminal cells and stars show SwG ducts. Scale bars = 25 μm; (c) Representative images of ACh- and ISO-induced sweat response in mouse paw pads. n = 3 for the measurements.

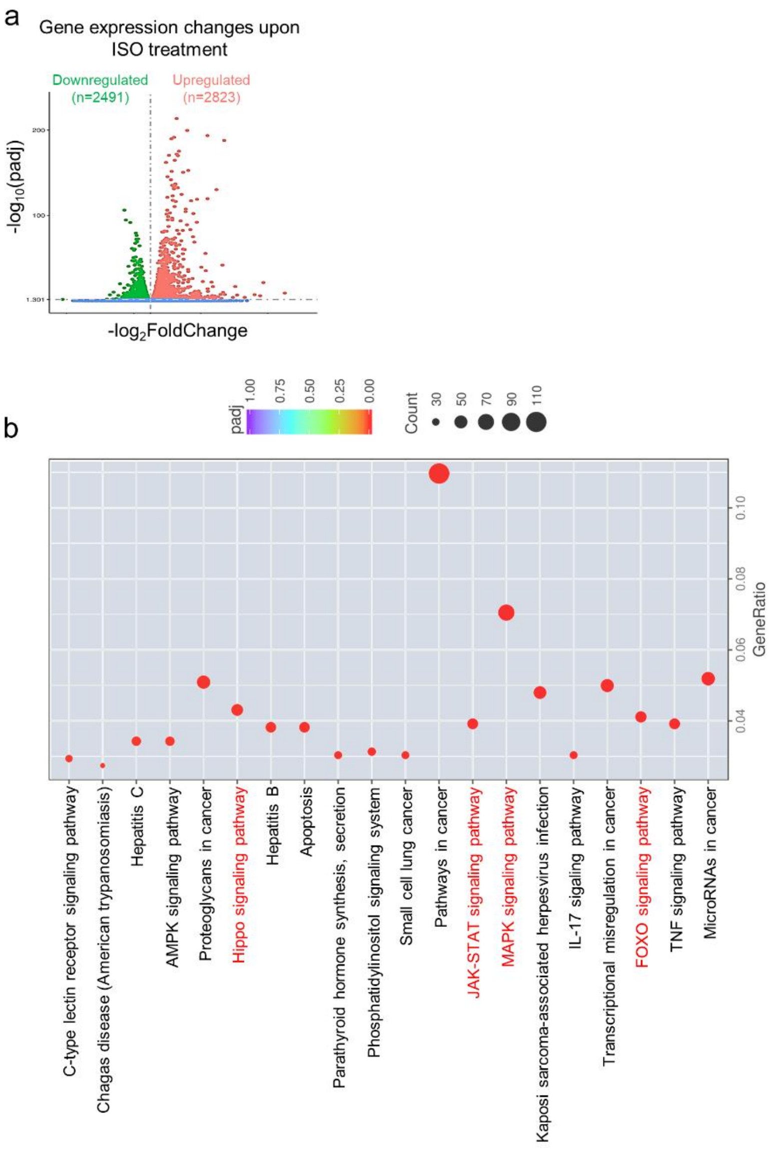

Figure S5 Gene Ontology functional analysis and KEGG pathway enrichment analysis of differentially expressed genes in HEKs. (a) Volcano plot showing the differentially expressed genes (DEGs) between ISO-treated versus untreated HEKs. Every dot represents a gene. A total of 5314 genes were differentially expressed in HEKs upon ISO administration (FDR ≤ 0.05 and $|FC| \geq 2.0$); (b) KEGG pathway enrichment analysis of DEGs. The rich factor represents the ratio of the DEG number to the total gene number in respective pathways. The top 20 positively enriched pathways are listed. Note that MAPK, JAK-STAT, Hippo, and FOXO signaling pathway were up-regulated in ISO-treated HEKs.

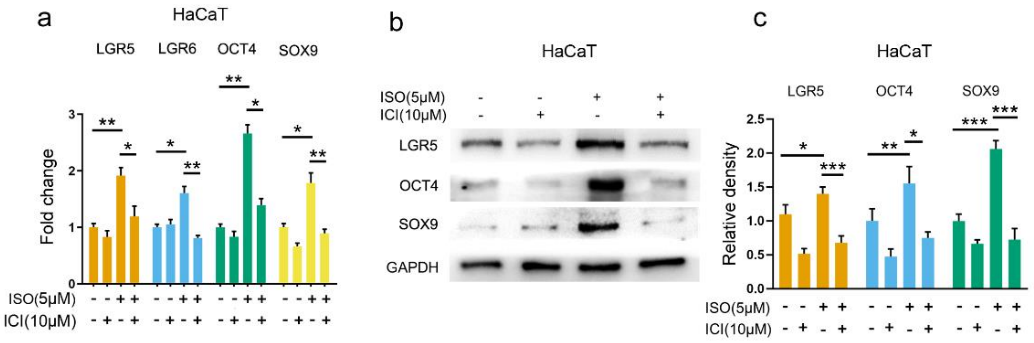

Figure S6 Beta2-AR activation upregulated stemness-associated gene expression in HaCaTs. (a) For β_2-AR blockage, starved HaCaT cells were pre-treated with 10 μM ICI-118,551 for 3 h. The cells were subsequently incubated in the presence or absence of 5 μM ISO for an additional 24 h. Then, relative mRNA expression levels of LGR5, LGR6, OCT4, SOX9 was assessed by quantitative RT-PCR (n = 3). The results were normalized to GAPDH expression; (b, c) Western blot analysis was performed to investigate the protein levels of LGR5, OCT4, SOX9 in HaCaT cells (n = 3) after switched to the medium containing 0, 5 μM ISO with or without ICI-118,551 (b). Quantitative western blot analysis of protein expression for LGR5, OCT4 and SOX9; (c). GAPDH was used as internal loading control. Data are mean ± SD. *P < 0.05, **P < 0.01, ***P < 0.001.

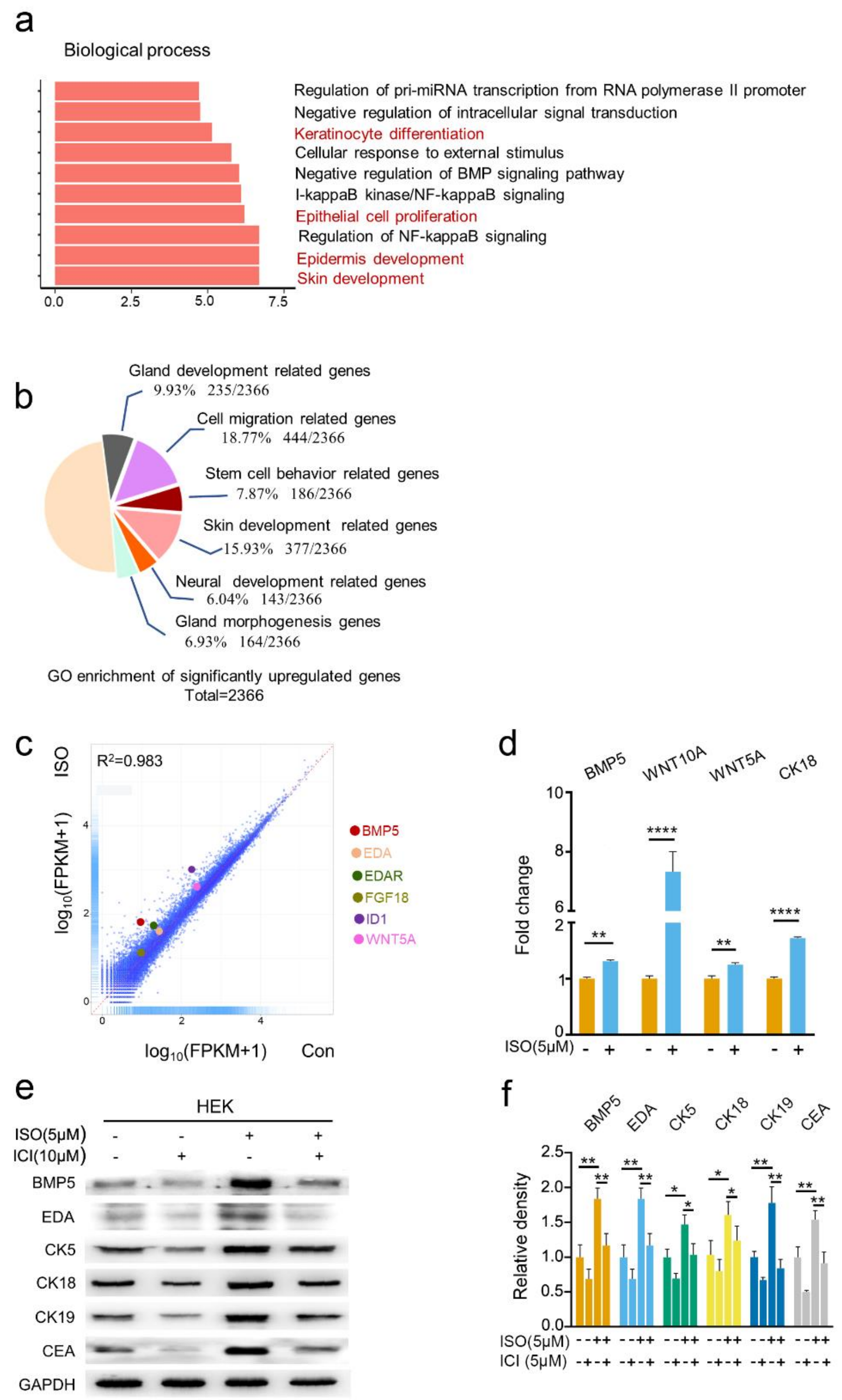

Figure S7 Beta2-AR activation upregulated specific SwG gene expression in HEKs. (a) Gene Ontology (GO) enrichment analyses of the 2366 upregulated genes in ISO-treated HEKs showing gene terms associated with keratinocyte differentiation, epithelial cell proliferation, epidermis development and skin development; (b) GO enrichment analysis of upregulated transcripts revealed

a set of genes associated with gland morphogenesis and development significantly enriched in ISO-treated versus untreated HEKs (FDR ≤ 0.05 and |FC| ≥ 2); (c) Scatter plot of RNA-seq transcriptome analyses showing SwG development-associated transcripts enriched in ISO-treated versus untreated HEKs (colored dots; FDR ≤ 0.05 and |FC| ≥ 2); (d) Starved HEKs were incubated in basic Epilife medium with or without ISO, and the relative mRNA expression of BMP5, WNT10A, WNT5A, CK18 was detected by quantitative RT-PCR; (e) Western blot analysis of protein levels of BMP5, EDA, CK5, CK18, CK19, and CEA in HEKs cultured in Epilife with or without ISO. ICI-118,551 was used to block β_2-AR activation; (f) Quantitative western blot analysis of protein expression for BMP5, EDA CK5, CK18, CK19, CEA. The results were normalized to GAPDH expression, n = 3. Data are mean ± SD. *P < 0.05, **P < 0.01, ****P < 0.0001 vs untreated group. ns, not significant.

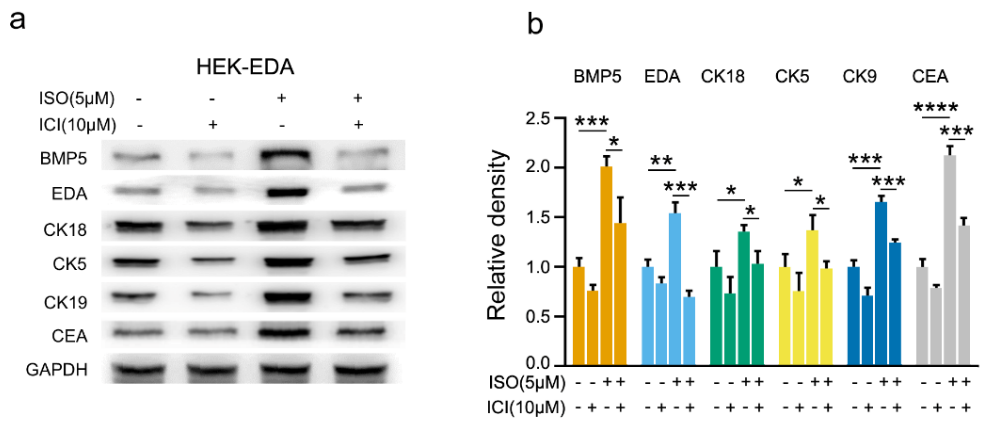

Figure S8 Activation of β$_2$-ARs upregulated of SwG-specific genes in iSwGCs. (a) Western blot analysis of BMP5, EDA, CK18, CK5, CK19, and CEA expression in HEK-EDA cells at day 8 after SGM treatment with or without ISO. For blockage of β$_2$-AR activity, a selective β$_2$-AR antagonist ICI-118,551 was added to the induction medium; (b) Quantification of western blot results showed that ISO treatment upregulated SwG gene expression, while addition of ICI-118,551 attenuated the effect of ISO on BMP5, EDA, CK18, CK5, CK19, CEA in HEK-EDA cells. GAPDH was used as internal loading control. n = 3. Data are mean ± SD. *$P < 0.05$, **$P < 0.01$, ***$P < 0.001$, ****$P < 0.0001$ vs untreated group. ns, not significant.

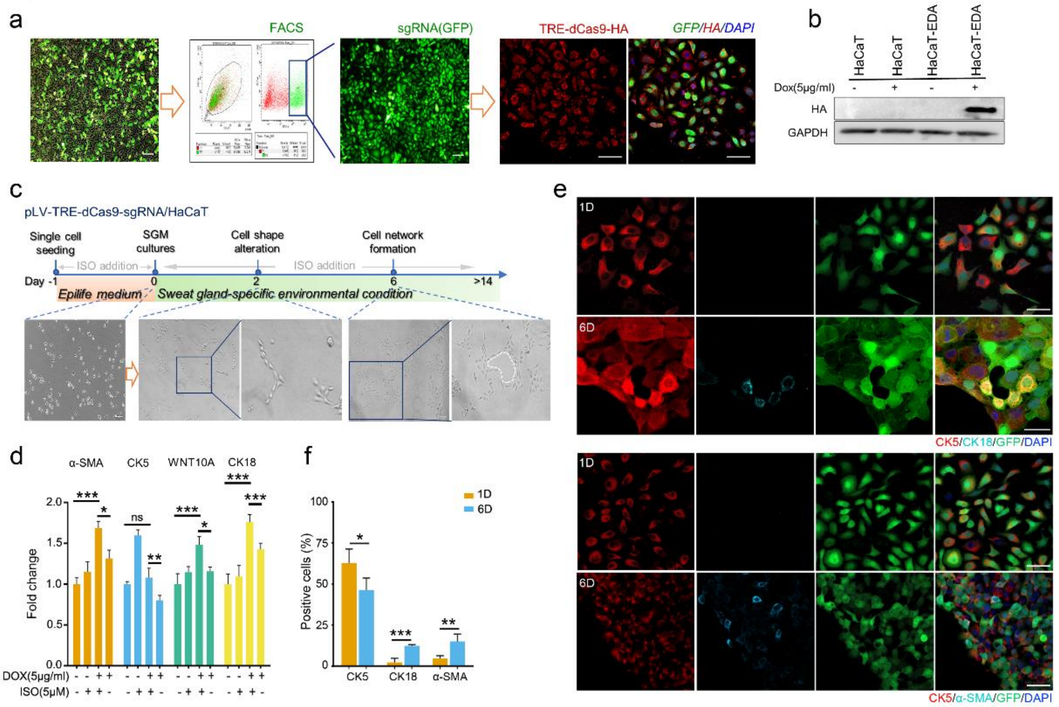

Figure S9 Generation of iSwGCs from HaCaT keratinocytes via CRISPR-mediated EDA activation together with optimized induction conditions. (a) Schematic of the protocol used for activating endogenous EDA in HaCaTs via CRISPR-dCas9 system. Scale bar =100 μm; (b) Western blot analysis of the expression of hemagglutinin (HA)-fused dCas9 protein in transfected HaCaT cells with or with doxycycline (Dox). n = 3; (c) Experimental design to assess the morphological changes of HaCaT-EDA cells after switched to SGM supplemented with 5 μg/ml Dox and 5 μM ISO. Scale bar =100 μm; (d) After incubation within SGM for 6 days, the transcriptional expression of α-SMA, CK5, WNT10A, CK18 was detected by quantitative RT-PCR. The results were normalized to GAPDH expression, n = 3; (e) Immunofluorescence assay of expression patterns of SwG markers in ISO-treated HaCaT-EDA cells after 1 or 6 days of SGM treatment n = 3, Scale bar = 50 μm; (f) Percentages of $CK5^+$, $CK18^+$, and $\alpha\text{-SMA}^+$ cells were shown in ISO-treated HaCaT-EDA cells after 1 or 6 days of SGM treatment. Quantification was done with 5 different fields from each of the 3 independent experiments. Data are mean ± SD. *$P < 0.05$, **$P < 0.01$.

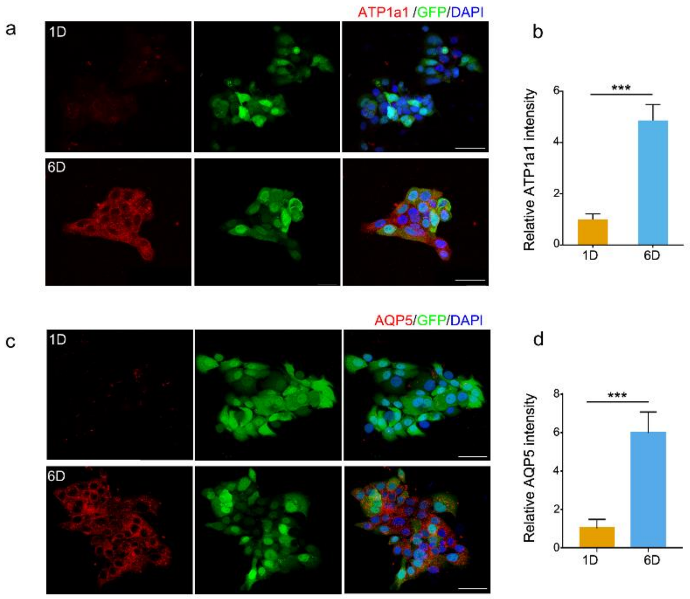

Figure S10 HaCaT-derived iSwGCs expressed SwG functional markers. (a) Immunofluorescence analysis of the expression of ATP1a1 in ISO-treated HaCaT-EDA cells after 1 or 6 days of SGM treatment. n = 3. Scale bar = 50 µm; (b) Fluorescence intensity quantification of ATP1a1 signals in ISO-treated HaCaT-EDA cells using ImageJ software; (c) Immunofluorescence analysis of time-course expression of AQP5 in ISO-treated HaCaT-EDA cells after 1 or 6 days of SGM treatment. n = 3. Scale bar = 50 µm; (d) Fluorescence intensity quantification of AQP5 signals in ISO-treated HaCaT-EDA cells using ImageJ software. Data are mean ± SD. ***$P < 0.001$.

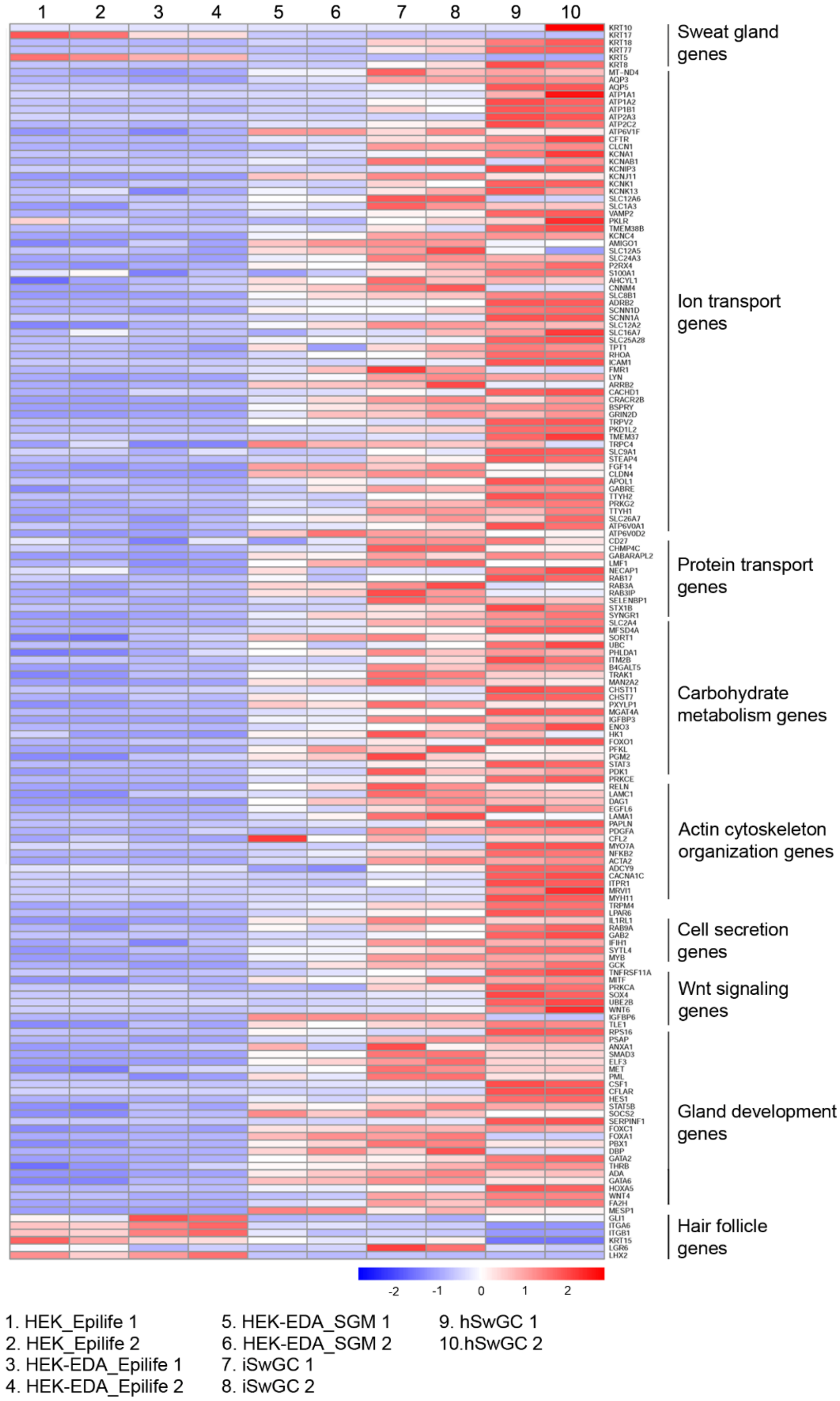

Figure S11 Heatmap of RNA-seq data displaying the expression of the indicated genes during iSwGC conversion. HEK-EDA_Epilife, Epilife-treated HEK-EDA cells; HEK-EDA_SGM, SGM-treated HEK-EDA cells; iSwGC, induced SwG cells; hSwGC, human SwG cells.

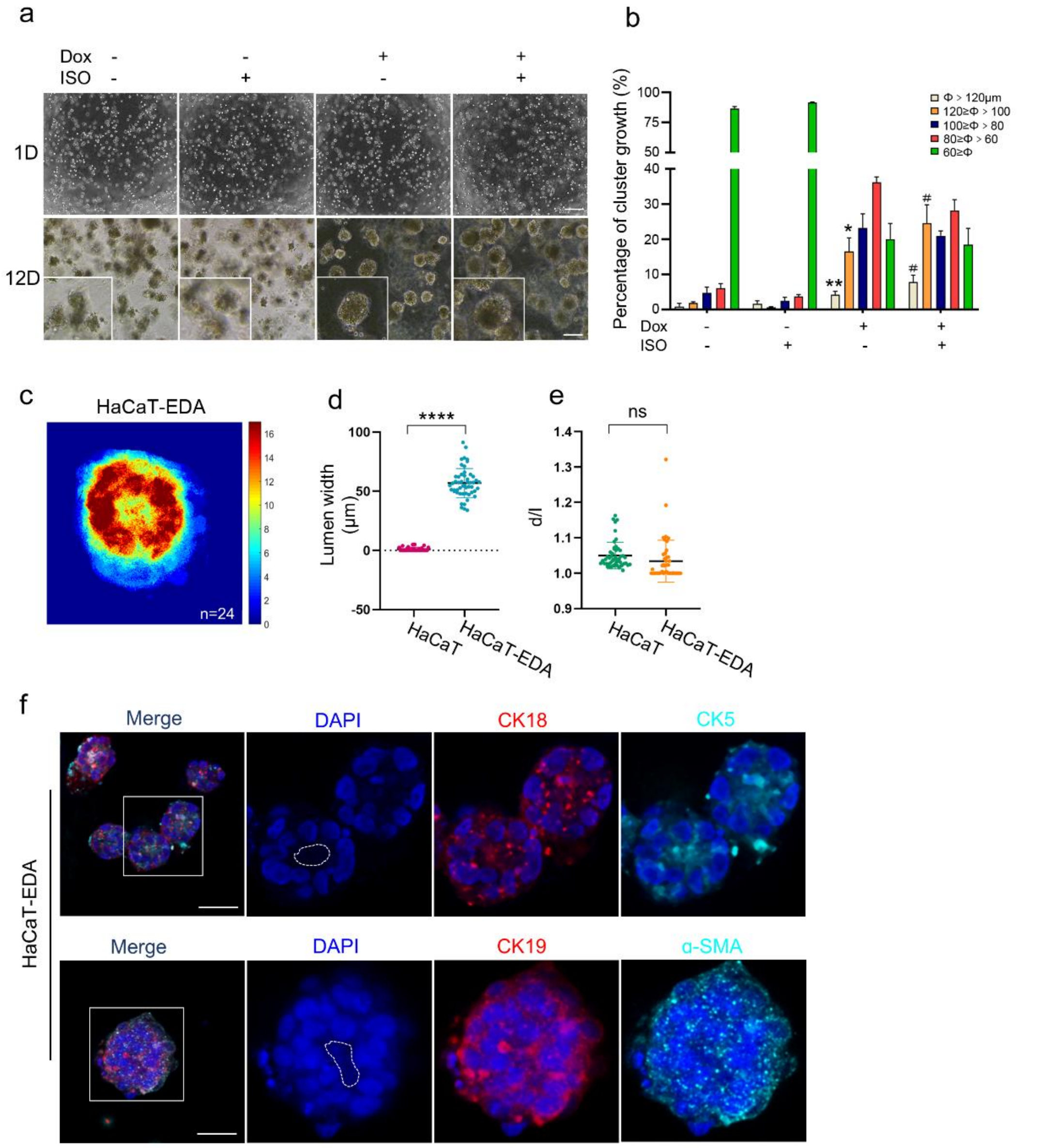

Figure S12 Generation of iSwGOs from reprogrammed HaCaTs. (a) Representative images showing the spheroid-forming capacity of HaCaT-EDA cells in SGM with or without ISO. Up row, Scale bar = 200 μm. Bottom row, Scale bar = 100 μm; (b) Quantification of spheroid-forming capacity of HaCaT-EDA cells under different induction conditions. Results are from a representative experiment (n = 3). Data are mean ± SD. *P < 0.05, **P < 0.01 vs control group. $^{\#}$P < 0.05 vs Dox-treated HaCaT-EDA cells; (c) Signal distribution acquired by confocal microscopy showing the quantification of luminal-containing organoids derived from reprogrammed HaCaTs (n = 24). Organoids with similar size were analyzed independently from 3 biological replicate; (d, e) Scatter plots representing the features of HaCaT-derived organoids. HaCaT cells were controls; (f) Im-

munofluorescence analysis of SwG markers in iSwGOs derived from reprogrammed HaCaTs. n = 3, Scale bar = 25 μm.

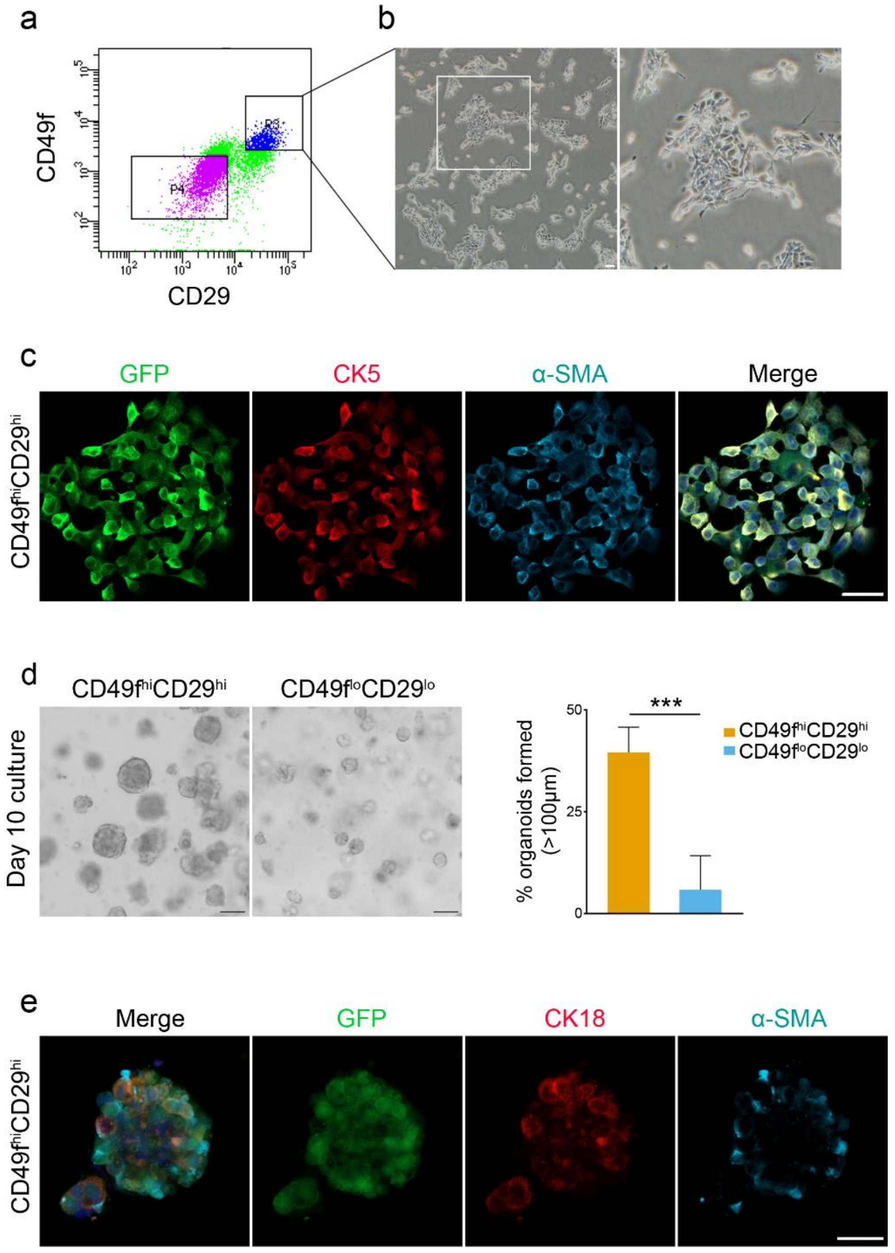

Figure S13 Generation of organoids from iSwGCs with myoepithelial identity. (a) Representative image showed gating for CD49f^{hi}CD29^{hi} fraction (P3 gate) in iSwGCs. P4 gate represented the CD49f^{lo}CD29^{lo} fraction in iSwGCs; (b) Phase contrast images of FACS-purified CD49f^{hi}CD29^{hi} iSwGCs. Scale bar = 100 μm. Insets, higher magnification of the boxed areas; (c) Immunofluorescence staining of myoepithelial-specific CK5 and α-SMA in the CD49f^{hi}CD29^{hi}-expressing cells. Scale bar = 50 μm; (d) Representative image showing the organoid growth capacity of CD49f^{hi}CD29hi-expressing cells. Scale bar = 100 μm. (e) Immunofluores-

cence staining of α-SMA and CK18 in the iSwGOs generated from $CD49f^{hi}CD29^{hi}$ cells. Scale bar = 50 μm. Data are mean ± SD representative of 3 independent experiments. ***P < 0.001.

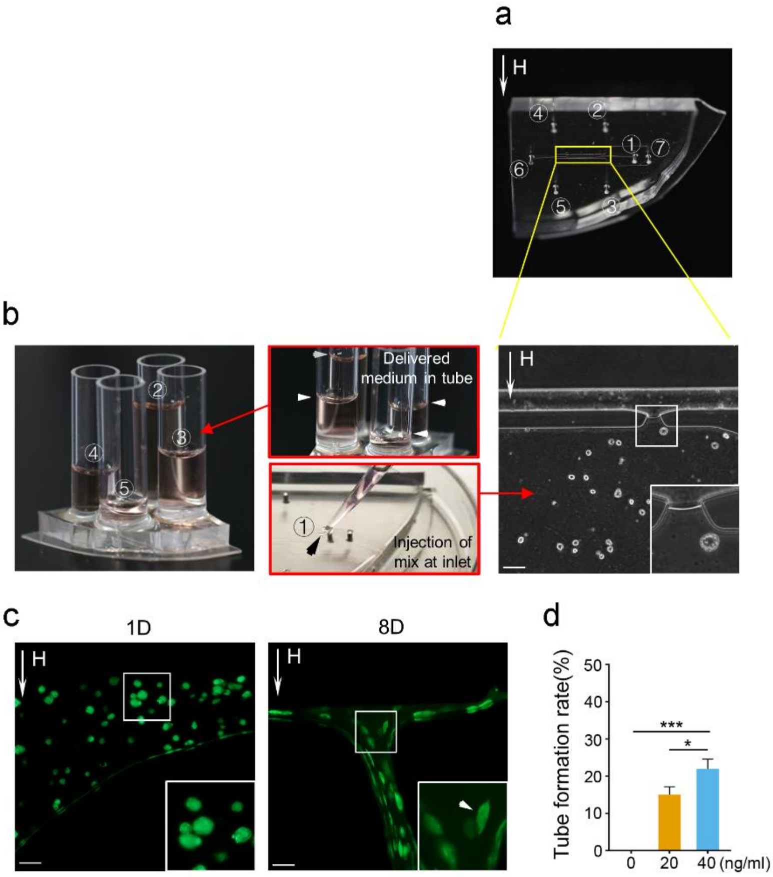

Figure S14 Self-organization of iSwGOs into tubular structures directed by bFGF gradients. (a) Schematic representation of the experimental setup. A bFGF concentration gradient was achieved in microfluidic devices owing to concentration differences between the microchip channel ②-④ and ③-⑤. Bottom row, scale bar = 75 µm; (b) Schematic description of the main steps for liquid handling in the microfluidic chip; (c) Representative images of the iSwGOs in the microfluidic chambers (left). After exposure to bFGF gradients for 8 days, organoids with bipolar shape were elongated along gradient direction (right). Scale bar = 75 µm, n = 3; (d) Quantification of the tube formation capacity of iSwGOs in response to different gradient concentrations. Data are mean ± SD representative of 3 independent experiments. *P < 0.05, ***P < 0.001.

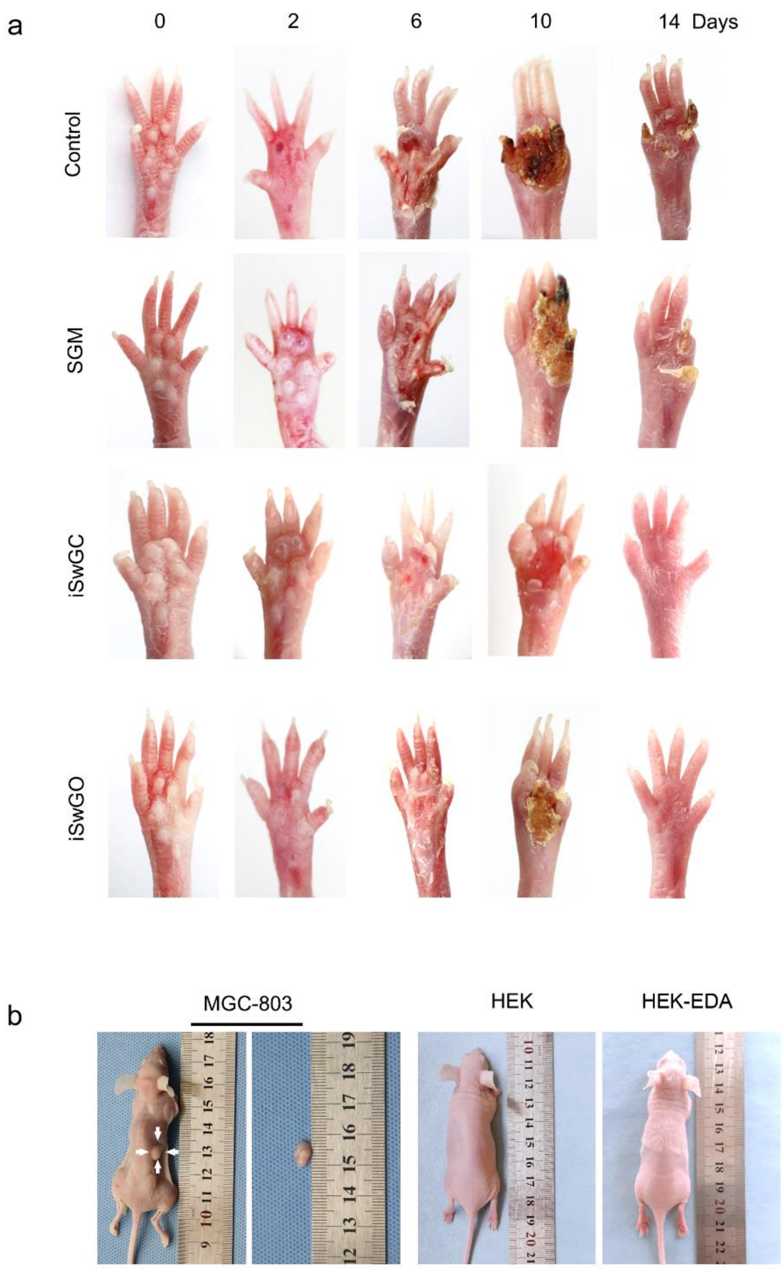

Figure S15 iSwGO transplantation promoted in-vivo skin wound healing. (a) Representative digital images of wounds at day 0, 2, 6, 10, and 14 in SGM-, iSwGC-, and iSwGO-treated mice. Mice treated with DMEM/F-12 were used as vehicle controls; (b) In-vivo safety assessment of HEK and HEK-EDA engraftment. MCG-803 gastric cell lines were used as positive controls. Cells were collected and injected subcutaneously at 10^7 cells per site. At 12 weeks after implantation, no tumors were seen in HEK and HEK-EDA-treated mice, whereas xenograft tumors were observed in MGC803 group. n = 3.

1. Supplementary methods

Constructs and Cell Transfection

Predesigned siRNAs directed to β_2-ARs (si-β_2-AR) and nontargeting control siRNAs were purchased from GenePharma, which were used at a concentration of 50 nM and contained the following sequences (5′ to 3′): si-β_2-AR-sense, CCACGACGUCACGCAGCAATT; si-β_2-AR-antisense, UUGCUGCGUGACGUCGUGGTT; negative control siRNA-sense, UUCUCCGAACGUGU CACGUTT; negative control siRNA-antisense, ACGUGACACGUUCGGAGAATT. HEKs were plated in basic Epilife medium (Invitrogen) in 6-well plates. When the cells reached a confluence of 60 - 70%, transfection was carried out by using INTERFERin in vitro siRNA/miRNA Transfection Reagent (Polyplus Transfection) according to the manufacturer's protocol. All transfections were carried out in triplicate and repeated at least 3 times.

In vivo tumorigenesis

To measure tumorigenicity in vivo in nude mice, cells including HEK, HEK-EDA, MCG-803 were collected and subcutaneously injected (10^7 cells per site) into five-week-old male BALB/c nude mice (Beijing HuaFuKang bioscience, Inc.). The mice treated with SwG-specific medium were used as blank controls. Tumor size was measured every 3 days. After 12 weeks, the mice were killed and tumor mass were weighted. All animal procedures were performed in accordance with institutional guidelines.

2. Table S1 Primer sequences used in the study

Primers	Sequences (5' to 3')
SOX9	Forward: AGACCTTTGGGCTGCCTTAT
	Reverse: TAGCCTCCCTCACTCCAAGA
LGR6	Forward: AGCCCTGTGAGTACCTCTTTG
	Reverse: CCACAGGAAATGCCAGTCAA
LGR5	Forward: CCTGCTTGACTTTGAGGAAGACC
	Reverse: CCAGCCATCAAGCAGGTGTTCA
OCT4	Forward: TATTCAGCCAAACGACCATC
	Reverse: TTGTTGTCAGCTTCCTCCAC
CK18	Forward: GTTGACCGTGGAGGTAGATGC
	Reverse: GAGCCAGCTCGTCATATTGGG
WNT5A	Forward: TCGACTATGGCTACCGCTTTG
	Reverse: CACTCTCGTAGGAGCCCTTG
WNT10A	Forward: AGATCGCCATCCACGAATGC
	Reverse: ATCTTGTTGCGAGTCTCCAGG
BMP5	Forward: CTCTACAATGCCATGACCAATGA
	Reverse: CGAGATAACTGTATGCGACGA
CK5	Forward: GGAGAAGGAGTTGGACCAGTCAAC
	Reverse: CTACCTCCGGCAAGACCTCCAC
α-SMA	Forward: ACTGCCTTGGTGTGTGACAA
	Reverse: CACCATCACCCCCTGATGTC
GAPDH	Forward: TGAAGGTCGGAGTCAACGGAT
	Reverse: CTGGAAGATGGTGATGGGATT
